# Personalized Web-Based Cognitive Rehabilitation Treatments for Patients with Traumatic Brain Injury: Cluster Analysis

**DOI:** 10.2196/16077

**Published:** 2020-10-06

**Authors:** Alejandro Garcia-Rudolph, Alberto Garcia-Molina, Eloy Opisso, Jose Tormos Muñoz

**Affiliations:** 1 Institut Guttmann Hospital de Neurorehabilitacio Badalona Spain; 2 Universitat Autònoma de Barcelona Bellaterra (Cerdanyola del Vallès) Spain; 3 Fundació Institut d’Investigació en Ciències de la Salut Germans Trias i Pujol Badalona Spain

**Keywords:** cluster analysis, traumatic brain injury, web-based rehabilitation

## Abstract

**Background:**

Traumatic brain injury (TBI) is a leading cause of disability worldwide. TBI is a highly heterogeneous disease, which makes it complex for effective therapeutic interventions. Cluster analysis has been extensively applied in previous research studies to identify homogeneous subgroups based on performance in neuropsychological baseline tests. Nevertheless, most analyzed samples are rarely larger than a size of 100, and different cluster analysis approaches and cluster validity indices have been scarcely compared or applied in web-based rehabilitation treatments.

**Objective:**

The aims of our study were as follows: (1) to apply state-of-the-art cluster validity indices to different cluster strategies: hierarchical, partitional, and model-based, (2) to apply combined strategies of dimensionality reduction by using principal component analysis and random forests and perform stability assessment of the final profiles, (3) to characterize the identified profiles by using demographic and clinically relevant variables, and (4) to study the external validity of the obtained clusters by considering 3 relevant aspects of TBI rehabilitation: Glasgow Coma Scale, functional independence measure, and execution of web-based cognitive tasks.

**Methods:**

This study was performed from August 2008 to July 2019. Different cluster strategies were executed with Mclust, factoextra, and cluster R packages. For combined strategies, we used the FactoMineR and random forest R packages. Stability analysis was performed with the fpc R package. Between-group comparisons for external validation were performed using 2-tailed t test, chi-square test, or Mann-Whitney U test, as appropriate.

**Results:**

We analyzed 574 adult patients with TBI (mostly severe) who were undergoing web-based rehabilitation. We identified and characterized 3 clusters with strong internal validation: (1) moderate attentional impairment and moderate dysexecutive syndrome with mild memory impairment and normal spatiotemporal perception, with almost 66% (111/170) of the patients being highly educated (*P*<.05); (2) severe dysexecutive syndrome with severe attentional and memory impairments and normal spatiotemporal perception, with 49.2% (153/311) of the patients being highly educated (*P*<.05); (3) very severe cognitive impairment, with 45.2% (42/93) of the patients being highly educated (*P*<.05). We externally validated them with severity of injury (*P*=.006) and functional independence assessments: cognitive (*P*<.001), motor (*P*<.001), and total (*P*<.001). We mapped 151,763 web-based cognitive rehabilitation tasks during the whole period to the 3 obtained clusters (*P*<.001) and confirmed the identified patterns. Stability analysis indicated that clusters 1 and 2 were respectively rated as 0.60 and 0.75; therefore, they were measuring a pattern and cluster 3 was rated as highly stable.

**Conclusions:**

Cluster analysis in web-based cognitive rehabilitation treatments enables the identification and characterization of strong response patterns to neuropsychological tests, external validation of the obtained clusters, tailoring of cognitive web-based tasks executed in the web platform to the identified profiles, thereby providing clinicians a tool for treatment personalization, and the extension of a similar approach to other medical conditions.

## Introduction

### Background

Every year, more than 50 million people worldwide experience a traumatic brain injury (TBI). It is estimated that about half the world’s population will have one or more TBIs in their lifetime. TBI is the leading cause of mortality in young adults and a major cause of death and disability across all ages worldwide, as recently reported in *The Lancet Neurology* [1]. Cognitive impairments due to TBI are the significant sources of morbidity in the affected individuals, their family members, and in the society. Disturbances in attention, memory, and executive functioning are the most common cognitive consequences of TBI at all levels of severity [2,3]. The clinical picture of TBI is characterized by a wide heterogeneity because of the nature and location of the injury [4]. Patients with TBI can show various combinations of motor, cognitive, behavioral, psychosocial, and environmental issues that have a huge impact on everyday activities [5], and these issues can greatly interfere with the effectiveness of rehabilitation interventions. It has been proposed that the efficacy of the rehabilitation would increase if programs moved from disease-centered to person-centered issues such that the rehabilitation is tailored to individual needs [6,7]. A number of studies have suggested that brain injury does not have any prototypical pattern of cognitive performance and outcome but may be best characterized by heterogeneity, both in regard to cognitive deficit and ultimate level of functioning [8]. TBI is an extremely heterogeneous disorder ranging from mild reversible conditions, often characterized as concussion, to severe massively destructive trauma, sometimes resulting in death. Saatman et al [9] highlighted the problem as follows: “The heterogeneity of TBI is considered as one of the most significant barriers to finding effective therapeutic interventions.”

### Clustering in TBI

TBI is a heterogeneous disease, and the mechanism/location of injury, premorbid functioning, secondary complications, and numerous other factors can influence cognitive performance [10]. As cognitive performance is a robust indicator of the current functioning and the prognostic outcome [11], it is critical to identify subgroups of patients who have distinct cognitive profiles that, in turn, can assist in treatment planning and patient care [12]. This can be empirically accomplished using cluster analysis, which is a multivariate classification technique that allows for statistical grouping of like cases into homogeneous subsets (or clusters) based on their similarity across one or more characteristics. Cluster analysis allows for the identification of homogeneous subgroups wherein cognitive heterogeneity is present based on the similarities in performance on neuropsychological tests.

Cluster analysis has been extensively applied in the study of TBI in the last 30 years [13-31]. Nevertheless, we have identified several common limitations such as the number of TBI patients that were clustered (<100 in many studies), the clustering approaches (only hierarchical clustering and k-means and not discussing other possible techniques), the specific implementation of such techniques (most of them restricted to only commercial products), as well as the lack of relation between the obtained clusters and rehabilitation tasks. The details are presented in Supplementary Material Table A1 (see Multimedia Appendix 1).

### Web-Based Cognitive Rehabilitation and Cluster Analysis

Cognitive rehabilitation has been playing an ever-increasing role in the treatment of patients with TBI who have cognitive deficits. The data gathered support the idea that improvements attributed to rehabilitation may generalize beyond task-specific skills [32]. Since the number of patients that could be eligible for this type of treatment is ever increasing, it is essential to develop new strategies that may improve access without elevating the costs to deliver such care [33]. The incorporation of computers and information technology-based systems in current clinical practice contributes to optimizing cognitive interventions, that is, their intensity, personalization, patient adherence, and quality of professional monitoring [34,35]. The types of cognitive rehabilitation programs that are the most effective in improving cognitive skills are still unclear [36]. Approaches that are designed to accommodate each individual’s cognitive strengths and weaknesses, offer instant item-specific feedback, and dynamically adapt the rehabilitation program accordingly appear to be the most effective, especially in populations with particular cognitive needs [37]. The objective of this study was to contribute to the personalization of web-based cognitive rehabilitation and to identify and characterize subgroups of patients who have distinctive profiles obtained from standard neuropsychological tests administered to patients before starting the rehabilitation.

### Main Characteristics of This Study

In the following subsections, we describe the main characteristics and specific objectives of this study.

#### Guttmann, NeuroPersonalTrainer

Guttmann, NeuroPersonalTrainer (GNPT)) is the web-based cognitive rehabilitation platform used in this study. GNPT addresses the desired features outlined in the previous section in the following manner.

1. It uses a baseline cognitive evaluation based on standardized neuropsychological tests to individualize the training regimen.
2. It continually adapts the difficulty level according to the subject’s performance by using an interactive-adaptive system.
3. It provides detailed graphic and verbal feedback after each rehabilitation task execution.

This study focuses on the baseline cognitive evaluation to individualize rehabilitation. Personalization of cognitive rehabilitation is accomplished by using a baseline cognitive evaluation, the results of which determine the individual content and the level of subsequent training for each participant. During rehabilitation, personalization is maintained by an adaptive feature that continually measures the subject’s performance, adapts the difficulty level of the training tasks, and provides detailed graphic and verbal performance feedback during and after each task. Because the rehabilitation regimen is designed based on the results of the cognitive evaluation and because the program continually adapts to each person’s strengths and weaknesses, it is unlikely that 2 participants can receive the same regimen with regard to the choice of tasks, amount, and intensity of rehabilitation in each cognitive domain.

#### Baseline Assessment: International Classification of Functioning Disability and Health

Baseline cognitive evaluation is performed in GNPT using the conceptual framework of the International Classification of Functioning, Disability and Health (ICF) [4]. The ICF belongs to a family of international classifications developed by the World Health Organization. ICF aims to provide a unified and standard language and framework for the description of health and health-related status. Direct punctuations obtained by patients in neuropsychological tests are mapped to the ICF 0-4 scale, representing the level of impairment, and they are expressed using ICF as complete disability (4), severe disability (3), moderate disability (2), mild disability (1), and no problem (0). The baseline assessment consists of the following 12 functions: categorization, divided attention, flexibility, inhibition, planning, selective attention, sequencing, spatial and temporal perception, sustained attention, verbal memory, visual gnosis, and working memory.

#### Individual Clustering Approaches

While numerous clustering algorithms have been published and new ones continue to appear, there is no single algorithm that has been shown to dominate other algorithms across all application domains [38]. Therefore, as an initial step, we proposed to study different clustering approaches in our application domain (the assessment instruments described in the previous section), and we tried different number of clusters (k). Clustering algorithms can be broadly divided into 2 groups: hierarchical and partitional (hierarchical has been applied in most publications presented in Table A1, Multimedia Appendix 1). In this study, we applied the following hierarchical and partitional algorithms: a hierarchical agglomerative algorithm AGNES (AGglomerative NESting), a hierarchical divisive DIANA (DIvisive ANAlysis), the classic k-means implementation, 2 partitional alternatives, that is, PAM (Partitioning Around Medoids) and CLARA (Clustering LARge Applications) [39], and a model-based clustering using the MClust software [40,41] (details are presented in Table A1, Multimedia Appendix 1).

#### Combined Approaches: Principal Component Analysis and Random Forest

As alternatives to individual clustering approaches, in this work, we present 2 combined approaches: principal component analysis (PCA) and random forest.

PCA can be viewed as a denoising method, which separates signal and noise: the first dimensions extract the essential parts of the information while the last ones are restricted to noise. Without the noise in the data, the clustering is more stable than the one obtained from the original distances. Consequently, if a hierarchical tree is built from another subsample of individuals, the shape of the top of the hierarchical tree remains approximately the same. PCA is thus considered as a preprocessing step before performing clustering methods [42]. PCA has been scarcely applied in previous research, as shown in Table A1 (Multimedia Appendix 1). In this study, we propose an integrated approach of PCA and hierarchical clustering.

Another recently proposed dimensionality reduction strategy is random forest. It consists of a collection or ensemble of classification trees, wherein each tree is grown with a different bootstrap sample of the original data. Each tree votes for a class and the majority rule is used for the final prediction. Random forests can be used in both supervised and unsupervised learning. In unsupervised random forests, the data is classified without a priori classification specifications. Synthetic classes are generated randomly and the trees are grown. Despite the synthetic classes, similar samples will end up in the same leaves of the trees owing to each tree’s branching process. The proximity of the samples can be measured and a proximity matrix is constructed. In this study, we propose the application of an unsupervised random forest integrated with the PAM clustering method [43].

### Study Objectives

We proposed to identify and characterize cognitive profiles in a web-based cognitive rehabilitation platform by using cluster analysis with the following specific aims:

1. Apply state-of-the-art cluster validity indices (CVIs) to different cluster strategies (hierarchical, partitional, and model-based) to identify meaningful classes.
2. Apply combined strategies of dimensionality reduction and clustering by using PCA and random forests to improve the obtained CVIs.
3. Characterize the identified profiles by using demographic and clinically relevant variables.
4. Study the external validity of the obtained clusters by considering 2 relevant aspects of TBI rehabilitation: functional independence measure (FIM) assessment (as well as Glasgow Coma Scale [GCS] for severity) at admission and rehabilitation and cognitive training tasks executed all along the rehabilitation process.

## Methods

### Participants

Our study consisted of patients with TBI who were admitted in the Rehabilitation Unit of the Acquired Brain Injury Department of a tertiary institution (Institut Guttmann, Spain). The period of the study was from August 2008 to July 2019.

This study was performed in accordance with the Declaration of Helsinki of the World Medical Association and approved by the ethics committee of the Clinical Research of this institution. Signed informed consent was obtained from every patient or their relatives after full explanation of the procedures. The inclusion criteria for the study were as follows: adult patients with the diagnosis of TBI and without any previous comorbidities leading to disability. Participants were excluded for illiteracy and inability to undergo formal cognitive evaluation for clinical reasons (eg, excessive sleepiness, bedridden patients, or uncontrolled sharp pain).

### Cognitive Evaluation: ICF Mapping

Initial cognition assessments used as input to cluster analysis were obtained through standardized administration of neuropsychological tests on admission; most of them were also applied to the state-of-the-art cluster analysis, as shown in Table A1 (Multimedia Appendix 1): Wisconsin Card Sorting Test, Barcelona Test, Rey Auditory Verbal Learning test, Wechsler Adult Scale III (digit span forward and backward), and Trial Making Test (Part A and Part B). All direct punctuations obtained by patients in each test were then mapped to the 0.4 ICF values. Details on the mapping of assessment instruments to ICF are presented in a previous study [44].

### Individual Cluster Analysis Approaches: Proposed Implementations

In this study, we took the 12 cognitive functions assessments (each one ranging from 0 to 4) as input to clustering techniques. For agglomerative hierarchical clustering, we applied the hclust function of the stats R package [45] and the AGNES function of the cluster [46] R package. For divisive hierarchical clustering, we applied the DIANA function of the cluster R package. The eclust function of the factoextra [47] R package was applied for the classic k-means implementation. The PAM function of the cluster R package was applied for PAM clustering, and similarly, the CLARA function of the same package was applied. For model-based clustering, the MClust [48] R package was applied.

### Combined Cluster Analysis Approaches: Unsupervised Random Forest Method

We proceeded using the following steps [43]:

1. The unsupervised random forest algorithm was used to generate a proximity matrix using the randomForest [49] R package.
2. PAM clustering of this first proximity matrix generated the initial classes.
3. A supervised random forest analysis of the initial classes allowed the calculation of out-of-bag error rates and the determination of the importance of the variables in relation to their contribution to accuracy in the classification.
4. Repeated the unsupervised random forest analysis with the most important variables to generate a second proximity matrix.
5. Repeated PAM clustering using the second proximity matrix to generate the new classes.
6. We then calculated the CVIs with the cluster.stats function of the fpc R package.

### Combined Approaches: PCA Method

We then considered an alternative approach, which combined dimensionality reduction and clustering: the hierarchical clustering on principal components (HCPC) function of the FactoMineR [50] R package. It involves the following steps:

1. Compute the principal components: PCA function for quantitative variables
2. Compute hierarchical clustering: It is performed using the Ward’s criterion on the selected principal components. Ward criterion is used because it is based on the multidimensional variance like PCA.
3. Choose the number of clusters based on the hierarchical tree: An optimal partitioning is proposed by HCPC to cut the hierarchical tree obtained using the AGNES technique.
4. Perform k-means clustering to improve the initial partition obtained from hierarchical clustering. The final partitioning solution, obtained after consolidation with k-means, can be (slightly) different from the one obtained with the hierarchical clustering.

### Performance Measures: Internal Validation and Stability

We then proposed to compare the internal validity (based only on the clustered data) of the resulting clusters based on the CVIs. These include average silhouette width [51], average Pearson gamma [52], entropy [53], Dunn index [52], and within-between cluster ratio (a higher metric of the former 3 statistics and a smaller within-between cluster ratio indicating a better fitting; eg, Clinical Cancer Research [54]). We focused especially on average silhouette width based on the conclusions in a recent review [55]. We applied the cluster.stats function of the fpc R package [56] to each of the proposed techniques for different number k of clusters, in order to obtain the CVIs. We focused on the average silhouette width by considering the following criteria [51]: 0.71-1.0, a strong structure has been found; 0.51-0.70, a reasonable structure has been found; 0.26-0.50, a weak structure has been found and could be artificial; and <0.25, no substantial structure has been found. In order to assess if the cluster holds up under plausible variations in the dataset (stability), our approach was to perform bootstrap resampling to evaluate the stability of a given cluster [57]. The cluster stability of each cluster in the original clustering is the mean value of its Jaccard coefficient over all the bootstrap iterations.

### Performance Measures: External Validation

As in previous publications presented in Table A1 (Multimedia Appendix 1), in order to validate any cluster solution, it is important to compare the resulting clusters on variables that were not included in the original clustering process [25]. Various demographic variables were examined for this purpose. Regarding statistical analysis, first, analysis of the homogeneity of variance by Levene’s test and normality of distribution by the Kolmogorov-Smirnov test were conducted. Chi-square tests were conducted for most of these variables because of their ordinal nature (eg, gender), whereas analyses of variance were performed with interval variables such as age. *P*<.05 was considered statistically significant. We included external variables that were described in previous studies such as gender, age, age ranges, education level, FIM [58], and severity at admission measured using the GCS. In Table A2 (Multimedia Appendix 1), we have included a detailed description of FIM and GCS.

A standard cognitive rehabilitation treatment in GNPT takes 2-5 months, which is distributed in 2-5 sessions a week, and each session is composed of 4-10 cognitive training tasks. GNPT integrates a set of about 100 web-based cognitive tasks, each of which mainly addresses one of the 12 functions described above. Typically, each patient executes a different number of tasks along with treatment and in a different order. For each execution, the patient obtains an immediate result (ranging from 0 to 100, as the percentage of compliance) [59].

## Results

### Sample Description

A final sample of 574 adult patients with TBI who performed web-based cognitive rehabilitation training in the GNPT platform were included in this study. The study was performed from August 1, 2008 to July 1, 2019. Of the 574 patients, 105 (18.3%) were women and 469 (81.7%) were men. Their distribution in the age ranges was as follows: 241 (42.0%) in the 17-30 years range, 259 (45.1%) in the 31-55 years range, and 74 (12.9%) in the >56 years range. With respect to the education level, of the 574 patients, 9 (1.6%) patients had completed primary education, 259 (45.1%) had completed secondary education, 205 (35.7%) completed tertiary education, and 101 (17.6%) completed post-tertiary education. The data of the severity of TBI at admission was available for 455 of the 574 patients (79.3%) by using the GCS, and the data were as follows: 44 (9.6%) had mild head injury, 57 (12.5%) had moderate head injury, and 354 (77.8%) had severe head injury.

### Baseline Clustering

**Figure 1:**
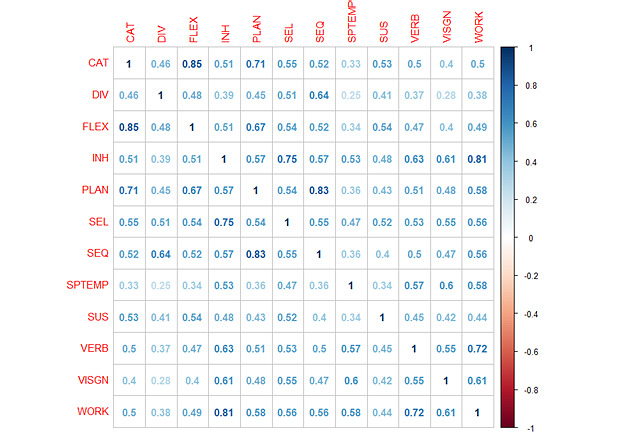
Correlogram of the initial set of cognitive variables. CAT: categorization; DIV, divided attention; FLEX: flexibility; INH: inhibition; PLAN: planning; SEL: selective attention; SEQ: sequencing; SPTEMP: spatiotemporal perception; SUS: sustained attention; VERB: verbal memory; VISGN: visual gnosis; WORK: working memory.

In order to run the implementations of the different algorithms presented in the Methods section, input parameters were selected as mentioned in previous state-of-the-art publications presented in Table A1 (Euclidean distance and Ward criteria). As the initial preprocessing phase, we performed Spearman correlation analysis by using the corrplot [60] R package in order to identify highly correlated variables. Figure 1 shows the correlation matrix among the 12 initial variables, which is colored according to the correlation coefficient. We observed the following 3 variables with *r*>0.80 and *P*<.001: flexibility, sequencing, and working memory. Therefore, we removed them for clustering.

Table 1 shows the internal validation results for different k values and for the 6 proposed clustering techniques.

**Table 1:** Internal validation of the proposed techniques for different number of clusters.

k for the different clusters		Average silhouette width	Pearson gamma	Entropy	Dunn index	Within-between cluster ratio
**AGNES (AGglomerative NESting)**						
	2	0.2947696	0.4446782	0.4193705	0.1466471	0.6827765
	3	0.3120659	0.6255316	0.9566682	0.1212678	0.5789247
	4	0.2664549	0.619668	1.055581	0.1280369	0.5791948
	5	0.2177597	0.6209335	1.173125	0.1324532	0.5722961
	6	0.2196451	0.5775517	1.445714	0.1336306	0.5558157
**DIANA (DIvisive ANAlysis)**						
	2	0.384397	0.5435043	0.5454738	0.09667365	0.6445731
	3	0.3427734	0.6395711	1.020228	0.1125088	0.5581218
	4	0.2918808	0.6340931	1.171331	0.1178511	0.5513584
	5	0.2603633	0.615897	1.311116	0.1178511	0.5523393
	6	0.2569563	0.547962	1.66604	0.1178511	0.5064622
**K-means**						
	2	0.3683991	0.534673	0.5815533	0.09712859	0.6497419
	3	0.3580276	0.6373444	1.0217	0.1125088	0.5584067
	4	0.3010858	0.5778943	1.342837	0.1212678	0.5373173
	5	0.2906528	0.5374244	1.602541	0.1212678	0.5092503
	6	0.2925957	0.5358799	1.759259	0.1360828	0.4798148
**PAM (Partitioning Around Medoids)**						
	2	0.3950954	0.541951	0.5103802	0.09407209	0.6434552
	3	0.3558112	0.6379774	1.022812	0.1125088	0.557843
	4	0.2912489	0.5634748	1.351603	0.1195229	0.5445628
	5	0.2801431	0.5516723	1.543992	0.1360828	0.5210802
	6	0.2889038	0.5414131	1.736641	0.1360828	0.4846027
**CLARA (Clustering LARge Applications)**						
	2	0.3917212	0.544271	0.524071	0.09667365	0.6431833
	3	0.3496284	0.6216692	1.038644	0.1125088	0.5626294
	4	0.292958	0.5662381	1.362308	0.1125088	0.5388617
	5	0.2809109	0.5496645	1.567395	0.125	0.5131381
	6	0.2890209	0.5298343	1.76809	0.125	0.4816711
**MClust**						
	2	0.2518519	0.4154374	0.6893485	0.1005038	0.7113782
	3	0.2897848	0.5629296	1.070214	0.1091089	0.5900842
	4	0.2389266	0.5382842	1.208638	0.1097643	0.592125
	5	0.2462358	0.5178902	1.506097	0.1118034	0.553266
	6	0.1889053	0.4791652	1.569551	0.1118034	0.5790436

### Random Forest: Classification Errors

We then calculated random forest classification with 2000 trees as input parameters, and we obtained the following overall out-of-bag errors for the different k values: 1.05% (k=3), 3.83% (k=4), and 5.23% (k=5). In Supplementary Material Table A3 (Multimedia Appendix 1), we present the confusion matrix for the different k values. When calculating variable importance, there was a loss of 20% in accuracy when removing the less important variable (visual gnosis) and 25% loss when removing inhibition, as shown in Supplementary Material Figure A2 (Multimedia Appendix 1). Therefore, no variable was removed, and we did not proceed to steps 4 and 5 of the methodology.

### PCA

Since FactoMineR uses a singular value decomposition algorithm, the PCA is calculated over the standardized correlation matrix, wherein a matrix of 40 uncorrelated components is obtained. Table S1 in Supplementary Material (Multimedia Appendix 1) shows the percentage of variance and the eigenvalues for the first 9 components of this matrix. The remaining components (31) correspond to a residual amount of variance. By selecting only the first 3 principal components, we reduced the dimensionality of the multivariate description so that the graphical representation and its subsequent interpretation were simplified. The first 3 principal components described 75.53% of the total variance. The first component described 55.04% of the variance, the second one described 13.42%, and the third component described 7.06%. In the case of the goodness of fit, we relied on the following metrics to verify the choice of the first 3 components: the root mean square of the residuals is 0.05 and the fit based upon off-diagonal values is 0.99.

We then ran the HCPC function with the following parameters: min=2, max=10, distance=Euclidean, criteria=Ward, and agglomerative hierarchical clustering.

When specifying min=2 and max=10 as parameters, HCPC identified the optimal k value maximizing the inertia gain. As shown in Supplementary Material Figure A3 (Multimedia Appendix 1), inertia gain dramatically decreased after the third class; therefore k=3 is the optimal partition proposed by HCPC.

### Internal Validation: Summary of the Results

When testing HCPC internal validation with the same indicators as presented in Table 1, we obtained the following CVIs: within-between ratio, 0.3706104; entropy, 0.9873104; Dunn index, 1.849996; Pearson gamma, 0.6511913; and average silhouette width, 0.515794. These CVIs clearly outperformed the CVIs presented in Table 1. For the individual approaches, the best average silhouette width was obtained by PAM for k=2 (0.395) and by k-means for k=3 (0.358). When the average silhouette width ranges from 0.26 to 0.50, the identified structure is weak and can be artificial. We focused especially on the average silhouette width, based on the conclusions in a recent CVI review [55], where 30 different indices with 720 synthetic and 20 real datasets were compared. A group of 10 indices was found to be the most recommended, with silhouette at the top in both synthetic and real datasets. Nevertheless, when considering the other CVIs in Table 1, the within-between ratio (the lower the better) HCPC was also the lowest, and Pearson gamma (the higher the better) was also higher for HCPC than any other in Table 1.

In relation to the random forest approach, when calculating variable importance, there was a loss of 20% in accuracy when removing the less important variable (visual gnosis) and 25% loss when removing inhibition. A previous study [43] removed variables leading to less than 5% loss in accuracy. In our case, no variable was removed, and therefore, we did not proceed to steps 4 and 5 of the methodology.

### Characterization of the Final Clusters

As presented in Table 2, the following clusters were found: cluster 1 (n=170), cluster 2 (n=311), and cluster 3 (n=93).

Table 2 shows statistically significant results for the education level of the participants as well as for all the involved cognitive functions. Analysis of cluster rationale indicated that cluster 1 is characterized by the highest level of education with almost 66% (66/170, 38.8% + 45/170, 26.5%) of its participants having tertiary or post-tertiary education. Meanwhile less than half of the participants in the other two clusters reach such educational levels: 49.2% (42/311, 13.5% + 111/311, 35.7%) of cluster 2 participants and 45.2% (14/93, 15.1% + 28/93, 30.1%) of cluster 3 participants. Furthermore, cluster 3 was characterized as complete impairment in all cognitive functions. Therefore, this cluster was characterized as very severe cognitive impairment. Meanwhile, cluster 1 presented mild impairment in working memory, visual gnosis, spatiotemporal perception, and inhibition and moderate impairment in categorization, divided attention, flexibility, planning, and sequencing. We characterized this cluster as highly educated, moderate attentional impairment, and moderate dysexecutive syndrome with mild memory impairment, and good spatiotemporal perception. Cluster 2 presented severe impairment in executive functioning (flexibility, categorization, and planning) and presented the highest degree of impairment in divided attention, as well as severe impairment in selective attention. Therefore, this cluster was characterized by severe dysexecutive syndrome with severe attentional and memory impairment and good spatiotemporal perception.

**Table 2:** Univariant analysis of the obtained clusters (N=574).

		Cluster 1, n=170	Cluster 2, n=311	Cluster 3, n=93	*P* value	
Age (years), mean (SD)		43.3 (14.4)	43.1 (15.2)	43.1 (14.5)		
**Gender, n (%)**					.84	
	Women	30 (17.6)	56 (18.0)	19 (20.4)		
	Men	140 (82.4)	255 (82.0)	74 (79.6)		
**Education level,** **n (%)**						<.05
	Post-tertiary	45 (26.5)	42 (13.5)	14 (15.1)		
	Primary	6 (3.53)	3 (0.96)	0 (0.0)		
	Secondary	53 (31.2)	155 (49.8)	51 (54.8)		
	Tertiary	66 (38.8)	111 (35.7)	28 (30.1)		
**Age range (years), n (%)**						.12
	17-30 years	61 (35.9)	131 (42.1)	49 (52.7)		
	31-55 years	86 (50.6)	138 (44.4)	35 (37.6)		
	56+ years	23 (13.5)	42 (13.5)	9 (9.68)		
**Baseline assessments, mean (SD)**						
	Categorization	2.14 (1.20)	3.72 (0.64)	4.00 (0.00)	<.001	
	Divided attention	2.34 (1.53)	3.94 (0.23)	4.00 (0.00)	<.001	
	Flexibility	2.12 (1.17)	3.58 (0.74)	4.00 (0.00)	<.001	
	Inhibition	0.64 (0.89)	2.34 (1.25)	4.00 (0.00)	<.001	
	Planning	2.09 (1.10)	3.56 (0.69)	4.00 (0.00)	<.001	
	Selective attention	1.58 (0.86)	3.29 (0.85)	4.00 (0.00)	<.001	
	Sequencing	2.06 (1.14)	3.57 (0.69)	4.00 (0.00)	<.001	
	Spatial and temporal perception	0.17 (0.44)	0.37 (0.64)	4.00 (0.00)	<.001	
	Sustained attention	1.35 (1.22)	3.03 (1.28)	3.71 (0.73)	<.001	
	Verbal memory	1.75 (1.01)	2.65 (0.95)	4.00 (0.00)	<.001	
	Visual gnosis	0.23 (0.59)	0.95 (1.30)	4.00 (0.00)	<.001	
	Working memory	0.73 (0.89)	1.95 (1.16)	4.00 (0.00)	<.001	

### External Validation

We performed twofold external validation: (1) by using demographic and clinical variables (age, gender, education level, age ranges) and then by using FIM and GCS evaluations at admission and (2) considering all cognitive tasks executed by the patients in GNPT during the period under study. We found no statistically significant differences when considering age, gender, or age ranges. The total number of available FIM assessments at admission was 439 of the original 574 participants (76.5%). Table 3 shows the number of participants, the mean, median, and IQRs for total FIM as well as the motor and cognitive subtotals for each cluster.

**Table 3:** Total functional independence measure, cognitive, and motor subtotals by cluster (N=439).

Measures		Cluster 1, n=138	Cluster 2, n=238	Cluster 3, n=63	*P* value
**Total functional independence measure**					<.001
	Mean (SD)	87.88 (33.55)	71.303 (38.07)	68.698 (39.26)	
	Median (Q1, Q3)	96.50 (65.25, 117.00)	73.000 (35.00, 108.00)	73.000 (28.00, 105.00)	
	IQR	18.00-126.00	18.00-126.00	18.00-126.00	
**Cognitive functional independence measure**					<.001
	Mean (SD)	26.96 (7.99)	22.58 (9.77)	21.452 (10.29)	
	Median (Q1, Q3)	29.00 (23.00, 33.00)	25.00 (15.00, 31.00)	22.00 (13.00, 30.00)	
	IQR	5.00-35.00	5.00-35.00	5.00-35.00	
**Motor functional independence measure**					<.001
	Mean (SD)	60.91 (27.175)	48.72 (30.02)	47.58 (30.47)	
	Median (Q1, Q3)	68.50 (40.00, 85.75)	48.00 (18.00, 79.00)	42.000 (14.00, 76.00)	
	IQR	13.00-91.00	13.00-91.00	13.00-91.00	

Regarding total FIM, patients in the 3 clusters required assistance for up to 25% of the tasks but cluster 3 was quite close to requiring assistance for 50% of the tasks. When considering the motor subtotal score with a maximum possible score of 91, patients in cluster 1 obtained 60.91, while cluster 2 obtained less than 50 and cluster 3 obtained 47.58. Regarding the cognition subtotal score (maximum score 35), cluster 1 was almost 30 while clusters 2 and 3 were close to 20.

In relation to GCS, the total number of available GCS assessments at admission was 455 (79.3%) of the original 574 participants. Table 4 shows the number of participants, mean, median, and IQRs for each cluster, and it shows the highest values for cluster 1, followed by cluster 2, and the lowest for cluster 3. Further, the IQR for cluster 3 ranged from 3 to 7, which was lower than that in clusters 1 and 2.

Regarding the second external validation, in GNPT, each task addresses a specific cognitive function. Table 5 shows the number of tasks for each function executed by cluster, with a total of 151,763 executions during the whole period under study.

**Table 4:** Total Glasgow Coma Scale measures by cluster (N=455).

Glasgow coma scale measures, *P*<.006	Cluster 1, n=136	Cluster 2, n=241	Cluster 3, n=78
Mean (SD)	7.19 (3.76)	6.40 (3.39)	5.50 (2.80)
Median (Q1, Q3)	7.00 (4.00, 10.00)	6.00 (4.00, 8.00)	4.50 (3.00, 7.00)
IQR	3.00-15.00	3.00-15.00	3.00-14.00

**Table 5:** Total task executions by cluster for all participating patients.

Task execution		Cluster 1, n=41,374	Cluster 2, n=89,577	Cluster 3, n=20,812	Total, N=151,763
**Functions (*P*<.001), n (%)**					
	Categorization	2137 (5.2)	4257 (4.8)	591 (2.8)	6985 (4.6)
	Divided attention	3673 (8.9)	7239 (8.1)	1038 (5.0)	11,950 (7.9)
	Flexibility	2470 (6.0)	5149 (5.7)	1642 (7.9)	9261 (6.1)
	Inhibition	2565 (6.2)	5605 (6.3)	1358 (6.5)	9528 (6.3)
	Planning	4636 (11.2)	9907 (11.1)	2114 (10.2)	16,657 (11.0)
	Selective attention	4776 (11.5)	12,460 (13.9)	4879 (23.4)	22,115 (14.6)
	Sequencing	3239 (7.8)	6067 (6.8)	1140 (5.5)	10,446 (6.9)
	Sustained attention	2907 (7.0)	9324 (10.4)	3206 (15.4)	15,437 (10.2)
	Verbal memory	9230 (22.3)	16,756 (18.7)	3162 (15.2)	29,148 (19.2)
	Visual gnosis	657 (1.6)	2830 (3.2)	75 (0.4)	3562 (2.3)
	Working memory	5084 (12.3)	9983 (11.1)	1607 (7.7)	16,674 (11.0)

**Figure 2:**
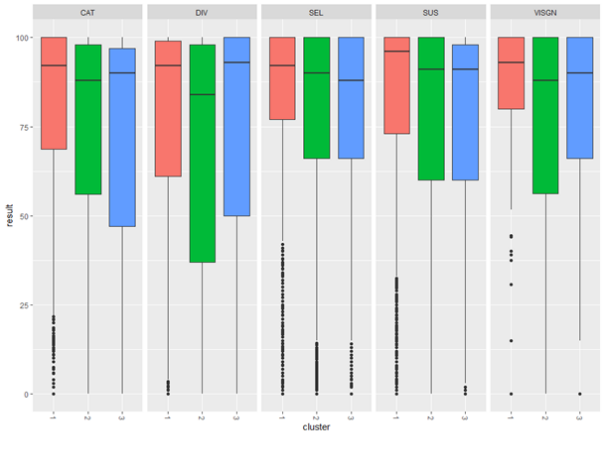
Tasks results boxplots for 5 cognitive functions: cluster 1 (red), cluster 2 (green), and cluster 3 (blue). CAT: categorization; DIV: divided attention; SEL: selective attention; SUS: sustained attention; VISGN: visual gnosis.

**Figure 3:**
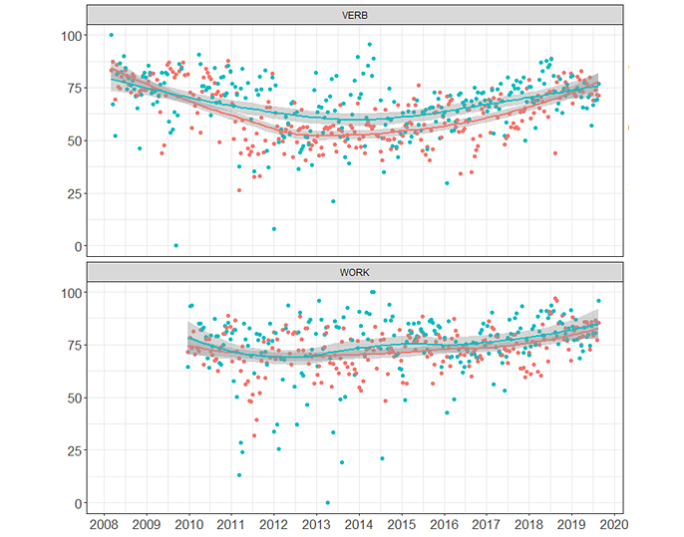
Mean values of the results in task executions summarized weekly, cluster 1 (blue) and cluster 2 (red). VERB: verbal memory; WORK: working memory.

Figure 2 shows the tasks result boxplots for 5 representative functions. Cluster 1 (at the left of each subplot) shows higher performance (punctuations closer to 100) than cluster 2, with cluster 3 showing lower punctuations. As shown in Table 2, for example, for the categorization function, the respective mean values for clusters 1, 2, and 3 were as follows: 2.14 (1.20), 3.72 (0.64), and 4.00 (0.00). The Figure 2 boxplots for the categorization function somehow reflect such different levels. Figure 3 represents the obtained results in every task execution for 2 functions: verbal memory and working memory. Verbal memory was the function with the largest number of executions, as shown in Table 5: 19.2% (29,148 of the total 151,763 task executions). In Figure 3, we present only cluster 1 (blue) and cluster 2 (red) in order to visually show their results, summarized weekly and plotted yearly during the whole period under study. Figure 3 shows that the working memory tasks have been integrated to the system in 2010, whereas verbal memory task executions started in 2008. For verbal tasks, cluster 1 patients outperformed cluster 2 during almost the whole period under study. Working memory tasks behave similarly, with a higher performance of cluster 2 patients.

### Stability

Values between 0.60 and 0.75 indicate that the cluster is measuring a pattern in the data, but there is no high certainty about which points should be clustered together. Clusters with stability values above 0.85 can be considered highly stable (they are likely to be real clusters). The obtained values by cluster were 0.7524206, 0.6647378, and 0.9910572. Therefore, there were 2 clusters with stability >0.75. As a rule of thumb, clusters with a stability value less than 0.60 should be considered unstable, which is not our case. Therefore, meaningful valid clusters as the ones identified in our study should not disappear if the data set is changed in a nonessential way. Nevertheless, it could also be of interest whether clusters remain stable under the addition of outliers; such cases should be individually considered by clinicians (eg, in case of the lowest GCS assessment values).

## Discussion

### Principal Findings

In this study, we proposed the application of cluster analysis to a chronic health condition in a GNU framework by using a set of publicly available R libraries (R-3.5.1) in the context of a web-based cognitive platform. We proposed 6 specific clustering techniques (ie, PAM, CLARA, AGNES, DIANA, k-means, and MClust) and 2 combined approaches (HCPC=PCA+AGNES and random forest+PAM) and evaluated them by using state-of-the-art CVIs. It is straightforward to apply both the individual techniques and the combined approaches to other acquired brain injury populations in the same web-based platform (GNPT) or in others. For example, in the Multimedia Appendix 1, we present an initial correlation analysis for patients who had an ischemic stroke that we will address in future work. We obtained the best CVIs with the combined HCPC=PCA+AGNES hierarchical clustering, with average silhouette over 52%; therefore, a *reasonable structure has been found.* We performed stability analysis, and clusters 1 and 2 were rated as 0.60 and 0.75, indicating that the clusters are measuring a pattern, and cluster 3 was rated as highly stable. We identified 3 clearly different profiles. Cluster 1 was characterized as highly educated, moderately distracted, with dysexecutive syndrome and good working memory. Cluster 2 was characterized as severe dysexecutive syndrome and severely distracted. Cluster 3 identified a group of patients with severe symptoms in all the involved functions. External validity in functional independence confirms this characterization by means of severity using GCS and functionality in the activities of daily living, especially when considering the motor FIM subtotal. When considering the performance in the cognitive tasks executed during the whole period, task results confirmed the identified profiles, with cluster 1 visual representation showing higher values during the whole period than cluster 2. Similar results were obtained when visualizing cluster 3.

### Clinical Implications

The actual GNPT implementation integrates an automatic therapy planning functionality, the intelligent therapy assistant (ITA) [61]. The ITA provides therapists with a recommended schedule of cognitive tasks to be executed by each patient during a given period of time. The recommendations provided by the ITA can always be manually modified by therapists according to their own clinical criteria. The ITA takes a predefined set of patient’s cognitive profiles as the starting point, which have been obtained using the baseline cognitive evaluation (mapped to ICF as described in the Methods section) as input to CA. When a new patient starts cognitive training in GNPT, the ITA dynamically assigns the patient to the appropriate cluster. The ITA then schedules different cognitive tasks during a user-defined rehabilitation period to the new patient, according to several criteria (eg, usage score, improvement score, clinical score) as described in previous studies. Therefore, the first clinical implication involves the ITA starting point to configure patients’ treatments. During therapy, when the patient executes a task (and obtains the result ranging from 0 to 100), GNPT automatically generates another version of the task with a higher or lower difficulty level—increasing the difficulty if the result was “too high” or decreasing the difficulty if the result was “too low” [62]. A second clinical implication involves linking cognitive profiles with performance in task execution. As shown in Figure 3, this allows therapists to identify patterns in performance, for example, results seem to be too close to 50 for cluster 2 in verbal memory tasks during the 2013-2016 period. The current clinical working hypothesis in relation to patient’s performance in GNPT tasks is that the optimal range of results is 65-85 [63]. Therefore, Figure 3 (top, verbal memory) suggests that difficulty levels in such tasks might have been too high for patients in cluster 2 during the 2013-2016 period. A more appropriate approach regarding the optimal range of results could be to consider such ranges to vary in relation to clusters. Therefore, a patient in cluster 1 would have a different optimal range than a patient in cluster 2. The next step is to consider the optimal range of the results depending on the cognitive profiles identified by cluster analysis (instead of considering a fixed optimal range as it is now). Future work should also include comparing ITA current cluster analysis results [61] with clusters 1, 2, and 3 obtained in this work for patients with TBI. The integration of cluster analysis as the initial phase of an ITA process also allows for a straightforward extension of a similar approach to other medical conditions, for example, patients who had a stroke, as we present in the Supplementary Material (Multimedia Appendix 1).

### Limitations of This Study

First, we conducted a single-center study; an advantage of this is that data were obtained and included by clinicians trained in neurological rehabilitation, and all patients were managed under the same TBI rehabilitation protocols. The GNPT platform is already integrated into the clinical practice of several acquired brain injury centers; nevertheless, their patients were not included in this analysis. A multicenter TBI study may include an initial preprocessing phase, wherein patients are grouped according to their initial GCS severity in order to avoid additional heterogeneity. Thereafter, cluster analysis techniques, as those proposed in this study, may be applied within such groups. External validation assessments, common to all participating centers, is also an important aspect to be addressed in this future multicenter study. Second, the health area studied belongs mainly to the urban population, with a small rural population or populations from other regions.

Third, our analysis lacked computerized tomography or magnetic resonance imaging examinations that describe the presence of contusion, hematoma, hemorrhage, ischemia, or other signs of parenchymal lesion on frontal, temporal, parietal, occipital, and cerebellar lobes or diffuse axonal injury. Fourth, our sample did not include any patient with missing data. All data used as input to cluster analysis are complete. Although there are several R packages addressing the subject (MICE, MissForest, HMISC), we decided to address the problem of missing data in a separate future analysis in order to consider not only the possible imputation strategies but also the reasons for missing data and include such reasons when characterizing the clusters. Fifth, our analysis did not include indicators of mental health or other comorbidities. Persons who experience TBI may have 1 or more preexisting medical comorbidities at the time of injury (eg, alcohol use and depression). Other medical conditions may occur simultaneously with TBI, such as orthopedic trauma, or these conditions may develop afterward as a direct consequence of the TBI such as epilepsy. Still, other medical comorbidities may begin months or years following injury in comparison to uninjured control groups. Studies have suggested that individuals with TBI have more than twice the rates of pain, growth hormone deficiency, insomnia, fatigue, new-onset stroke, urinary incontinence, and epilepsy [64]. Therefore, we aim to include comorbidity analysis in future research studies.

### Comparison with Prior Work

We have worked with public GNU libraries, as opposed to the state-of-the-art publications presented in Table A1, wherein most techniques were implemented using commercial packages [15-18,20-23,25-27,29-31]. Previous research presented in Table A1 applied clustering techniques in a batch mode as desktop applications. In our case, the work was integrated in the context of a web-based cognitive training platform. Our baseline assessment consisted of 12 cognitive functions, thereby allowing for a comprehensive description of the patient’s profiles, involving cognitive aspects addressed by such different functions, ranging from visual attention to gnosis. Meanwhile, previous clustering research presented in Table A1 addresses specific functions—only one of them in most cases: memory [14,16,18-21,24-26,30], executive functions [17,21,31], or attention [22]. We have proposed different clustering techniques and applied state-of-the-art CVIs to all of them. We have taken advantage of the web-based platform by increasing the number of participants, whereas in only 3 of the 20 studies in Table A1, n is larger than 300 [20,25,30]. We have included the whole set of cognitive tasks performed by all participants as part of the external validation during the whole period under study (more than 150,000 task executions). We have visually mapped such executions to the obtained clusters along time. To the best of our knowledge, the linking of specific rehabilitation tasks to the obtained clusters has not been yet performed in the state-of-the-art publications presented in Table A1.

### Conclusions

Cluster analysis in web-based cognitive rehabilitation treatments allows for identifying and characterizing strong patterns of response to neuropsychological tests, externally validating the obtained clusters by using important aspects of TBI rehabilitation such as severity or functional independence in activities of daily life, tailoring cognitive web-based tasks available in the web platform to the identified profiles by providing clinicians a tool for treatment personalization, which were not addressed in previous traditional cluster analyses, and straightforward extension of a similar approach to patients with other medical conditions, for example, for patients who have had a stroke.

## Appendix

Multimedia Appendix 1 Previous studies of cluster analysis of traumatic brain injury based on neuropsychological tests; R code and plots of applied techniques, random forest approach details, and principal component analysis approach details.

## Abbreviations

AGNES: AGglomerative NESting
CLARA: Clustering LARge Applications
CVI: cluster validity index
DIANA: DIvisive ANAlysis
FIM: functional independence measure
GCS: Glasgow Coma Scale
GNPT: Guttman, NeuroPersonalTrainer
HCPC: hierarchical clustering on principal components
ICF: International Classification of Functioning, Disability and Health
ITA: intelligent therapy assistant
PAM: Partitioning Around Medoids
PCA: principal component analysis
TBI: traumatic brain injury
