# Supplementary material for: Personalized Web-Based Cognitive Rehabilitation Treatments for Patients with Traumatic Brain Injury: Cluster Analysis
Source: JMIR Med Inform. 2020 Oct 6;8(10):e16077. doi: 10.2196/16077 (PMC7576523; doi:10.2196/16077)

SUPP MATERIAL

Table A1. Cluster Analysis studies in TBI based on neuropsychological tests

| Targets | Tests | Year Reference | n | Technique | k |
| --- | --- | --- | --- | --- | --- |
| VIQ, PIQ | WAIS-R | 1990 [13] | 93 | CLUSTAN hierarchical | 6 |
| Memory | CVLT | 1992 [14] | 70 | Hierarchical, Euclidean, Ward’s method | 3 |
| PIQ, Memory, Attention, visuospatial | WAIS-R, Stroop, RAVLT, COWAT, Trails A, WCST PE | 1993 [15] | 47 | Hierarchical, Euclidean, Ward’s method | 5 |
| Memory | CVLT | 1994 [16] | 65 | Hierarchical, Euclidean, Ward’s method | 4 |
| Executive functions | IHCT | 1995 [17] | 87 | Hierarchical, Euclidean, Ward’s method | 4 |
| Memory | CVLT | 1996 [18] | 88 | k-means +hierarchical Ward’s, EML, WPGMA, UPGMA | 5 |
| Memory | CVLT | 1999 [19] | 150 | FASTCLUST Hierarchical, Euclidean, Ward’s method | 4 |
| Memory | CVLT and WMS–R | 2001 [20] | 301 | Ward’s method of hierarchical cluster +  k-means | 5 |
| Memory | CVLT | 2002 [21] | 160 | Ward’s method of hierarchical cluster +  k-means | 2 |
| Attention | STROOP, Tower of Hanoi, PASAT, 6E | 2003 [22] | 92 | Hierarchical + non-hierarchical seed points | 3 |
| VCI, POI, WMI, PSI | WAIS-III | 2003 [23] | 166 | FASTCLUST Hierarchical, Euclidean, Ward’s method + kmeans | 3 |
| Memory | CVLT | 2006 [24] | 175 | FASTCLUST Hierarchical, Euclidean, Ward’s method + kmeans | 4 |
| Memory | CVLT-II | 2008 [25] | 1,087 | FASTCLUST Hierarchical, Euclidean, Ward’s method + kmeans | 6 |
| Memory | CVLT-II | 2010 [26] | 223 | FASTCLUST Hierarchical, Euclidean, Ward’s method + kmeans | 6 |
| Attention Executive functions | TMT-A, TMT-B | 2013 [27] | 78 | Hierarchical, Euclidean, Ward’s method + kmeans | 3 |
| VCI, POI, WMI, PSI | WAIS–III | 2013[28] | 220 | Hierarchical, Euclidean, Ward’s method + kmeans | 4 |
| Executive functions | WCST, TMT-B, Hayling | 2015 [29] | 84 | Hierarchical, Euclidean, Ward’s method + kmeans | 3 |
| Memory | RAVLT, FAS, TMT A,B, | 2015 [30] | 491 | VARCLUS (SAS) Hierarchical Euclidean +Principal Components Analysis | 4 |
| Executive functions | CTMT | 2018 [31] | 121 | Hierarchical, Euclidean, Ward’s method + kmeans + DFA | 3 |

Hierarchical clustering algorithms recursively find nested clusters either in agglomerative mode (starting with each data point in its own cluster and merging the most similar pair of clusters successively to form a cluster hierarchy) or in divisive (top-down) mode (starting with all the data points in one cluster and recursively dividing each cluster into smaller clusters).

Compared to hierarchical clustering algorithms, partitional clustering algorithms find all the clusters simultaneously as a partition of the data and do not impose a hierarchical structure. Input to a hierarchical algorithm is an n _ n similarity matrix, where n is the number of objects to be clustered. On the other hand, a partitional algorithm can use either an n x d pattern matrix, where n objects are embedded in a d-dimensional feature space, or an n x n similarity matrix.

The underlying idea of model-based clustering is that the observed data in a population actually come from several subpopulations, which can be modeled separately. Then using finite mixture models, the overall population is modeled as a mixture of these subpopulations

Table A2. Cutoff values and description of external validation assessments

| **Assessment** | **Cutoff values** | **Description** |
| --- | --- | --- |
| Functional Independence Measure (FIM) | 18 | Complete dependence |
|  | 19-60 | modified dependence (assistance for up to 50% of tasks); |
|  | 61-103 | modified dependence (assistance for up to 25% of tasks) |
|  | 104-126 | complete/ modified independence |
| Glasgow Coma Scale (GCS) | ≤ 8 | Severe Head Injury |
|  | 9-12 | Moderate Head Injury |
|  | 13-15 | Mild Head Injury |

Functional independence after TBI is an important outcome and necessary guide for decision-making on part of treatment-team and family members. FIM evaluates the ability of patients with functional restrictions of various causes; including TBI, it quantitatively evaluates the care demand of a person for performing motor and cognitive tasks of daily living providing separate motor and cognitive subtotals.

In this study we also propose to use cognitive training tasks as external validation, trying to identify different patients’ profiles leading to different performance in tasks executions

BASELINE CLUSTERING APPROACHES

**Hierarchical clustering**

#DIVISIVE HIERARCHICAL

dist.eu<-dist(dat, method = "euclidean", diag = FALSE, upper = FALSE, p = 2)

#diana= DIvisive ANAlysis Clustering

hc.divisive <- diana(as.matrix(dist.eu), diss = FALSE, keep.diss = TRUE)

plot(hc.divisive, main = "Divisive")


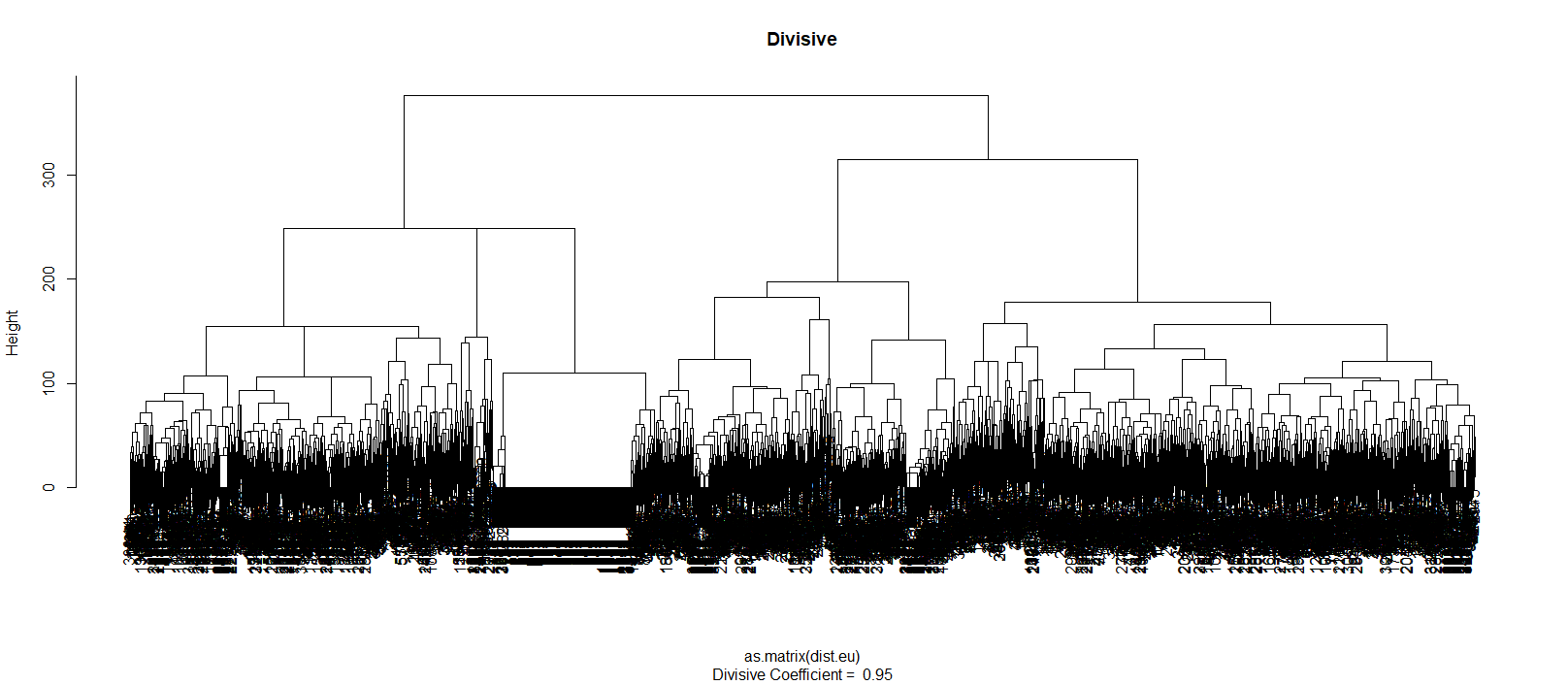


#AGGLOMERATIVE HIERARCHICAL HCLUST complete linkage

dist.eu<-dist(dat, method = "euclidean", diag = FALSE, upper = FALSE, p = 2)

# p = 1 is equivalent to the Manhattan distance and the case where p = 2 is equivalent to the Euclidean distance.

aggl.clust <- hclust(dist.eu, method = "complete")

plot(aggl.clust, main = "Agglomerative")


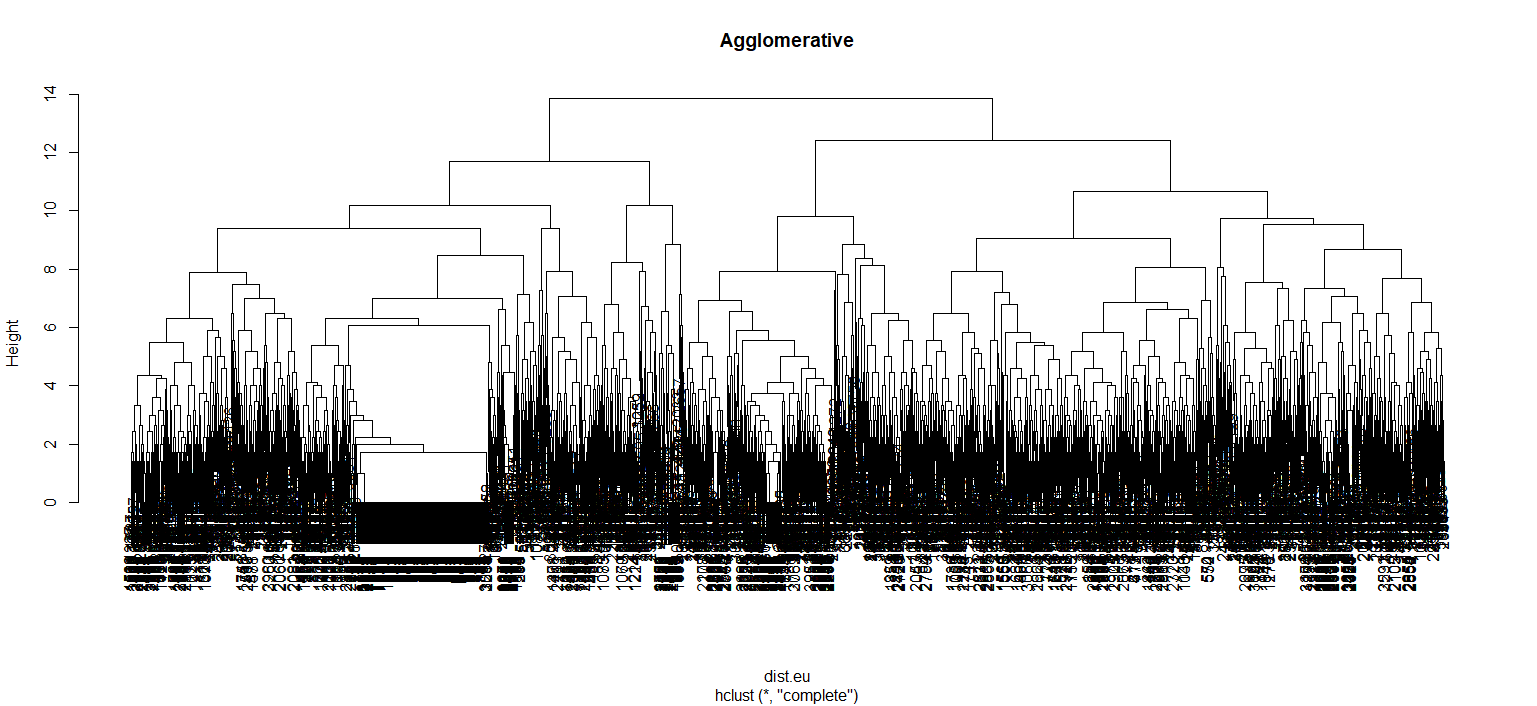


k-MEANS

km.res <- eclust(dat, "kmeans", k = 2, nstart = 25, graph = FALSE)

#nstart option attempts multiple initial configurations and reports on the best one. For example, adding nstart=25 will generate 25 initial random centroids and choose the best one for the algorithm

# k-means group number of each observation

km.res$cluster

# Visualize k-means clusters

fviz_cluster(km.res, geom = "point", frame.type = "norm")


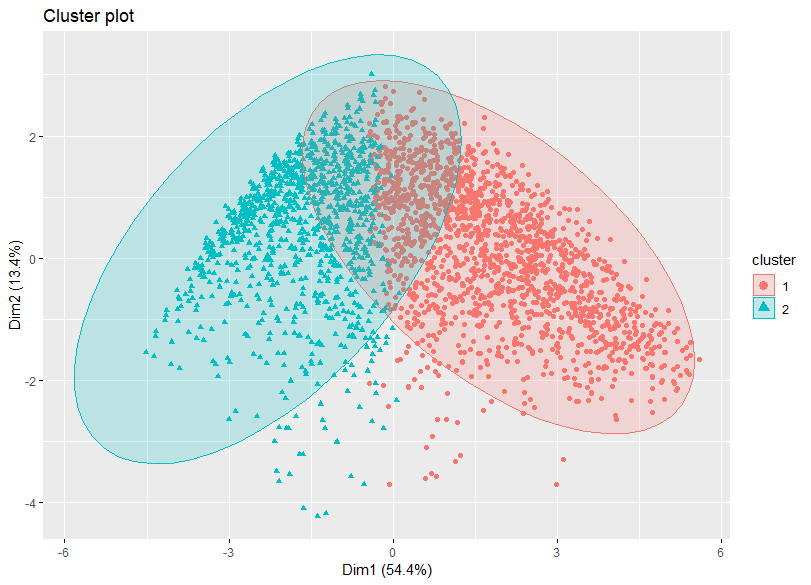

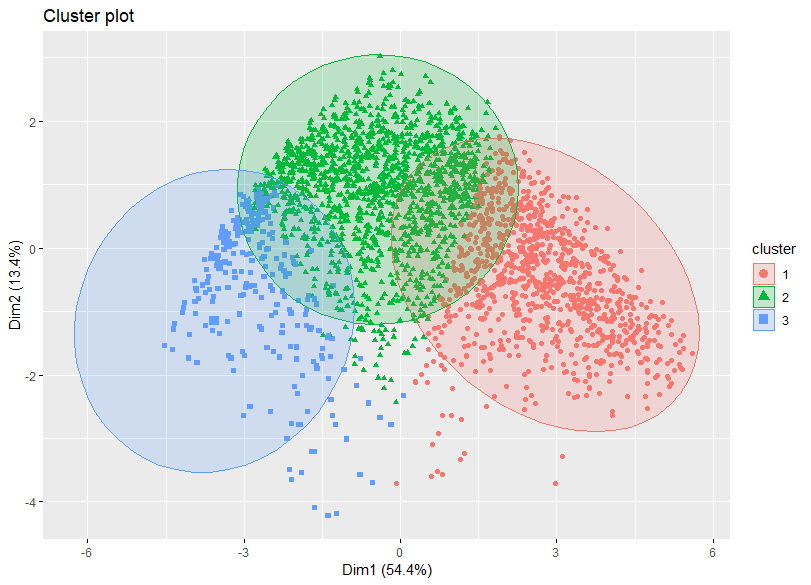


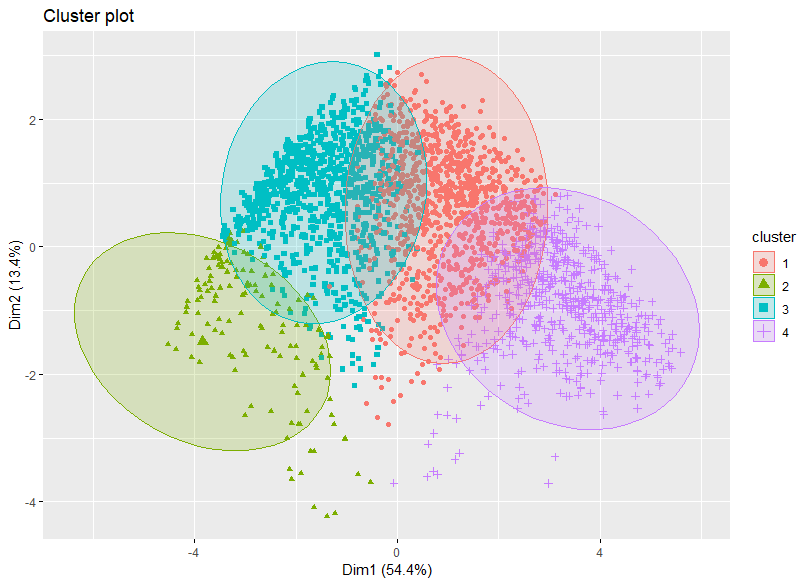

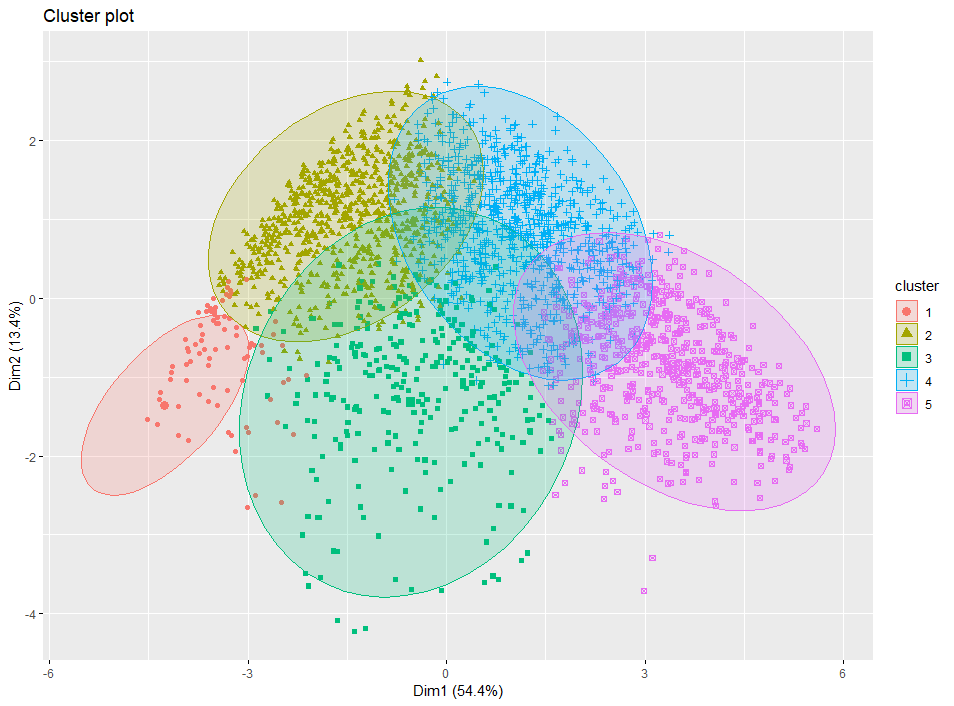


PAM

Partitioning (clustering) of the data into k clusters “around medoids”, a more robust version of K-means.

km<- pam(dat, 2, metric = "euclidean", stand = FALSE)

#stand= if TRUE, then the measurements in x are standardized before calculating the dissimilarities

fviz_cluster(km, geom = "point", ellipse.type = "norm")

dist.eu<-dist(dat, method = "euclidean", diag = FALSE, upper = FALSE, p = 2)

km_stats <- cluster.stats(dist.eu, km$cluster)


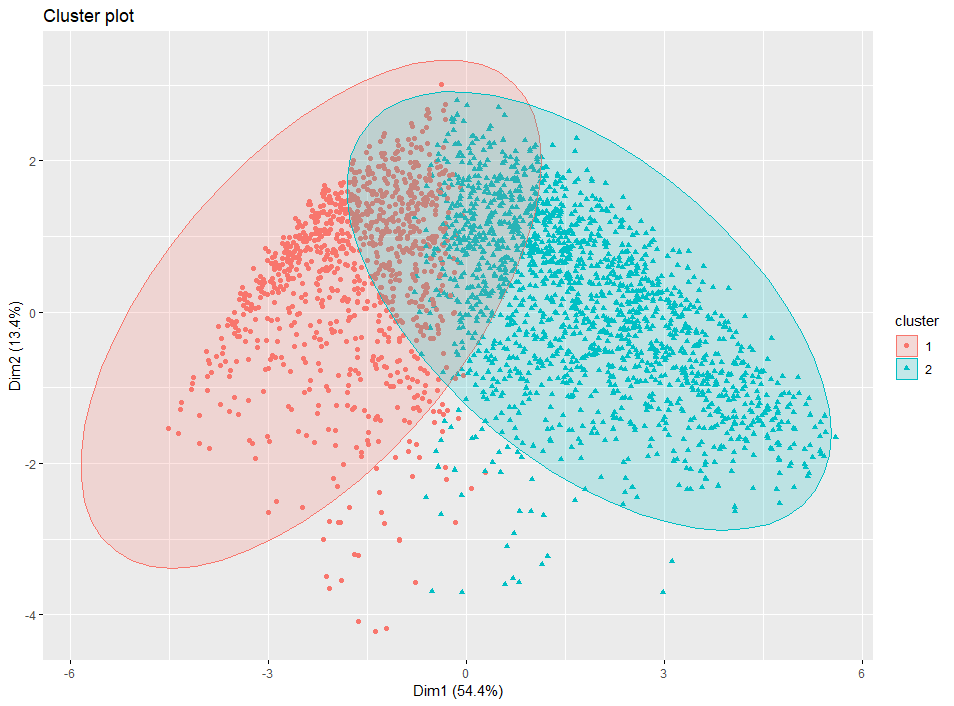

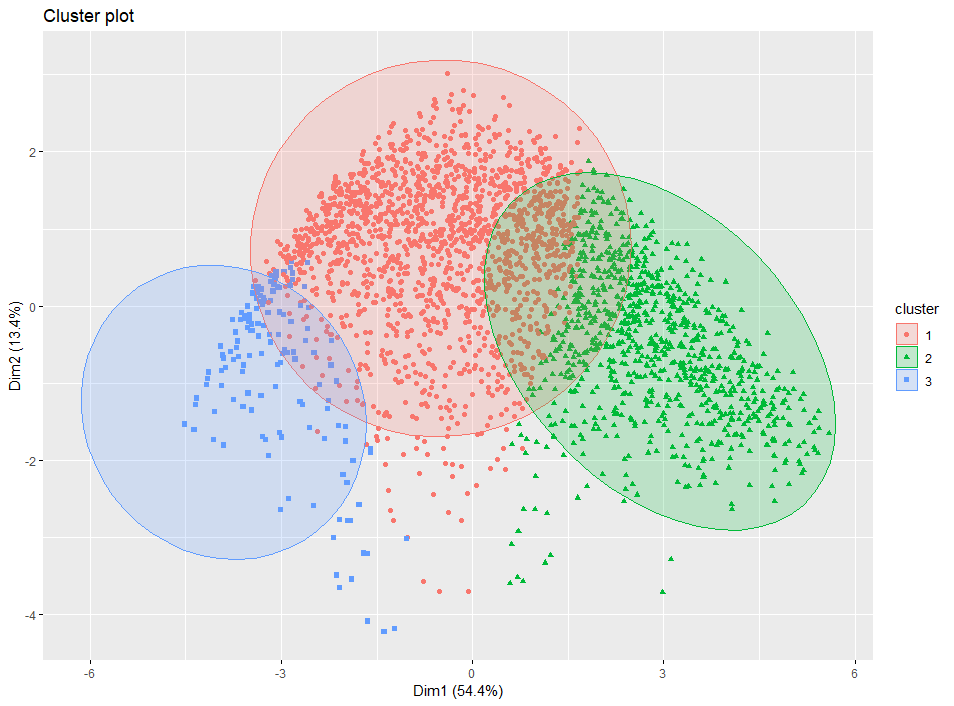


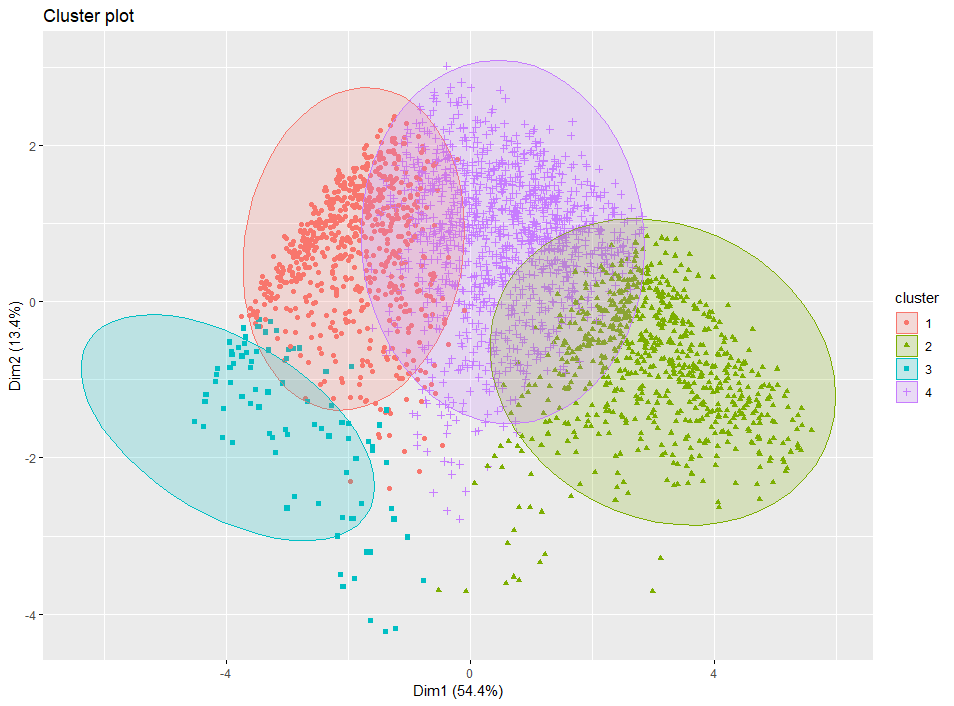

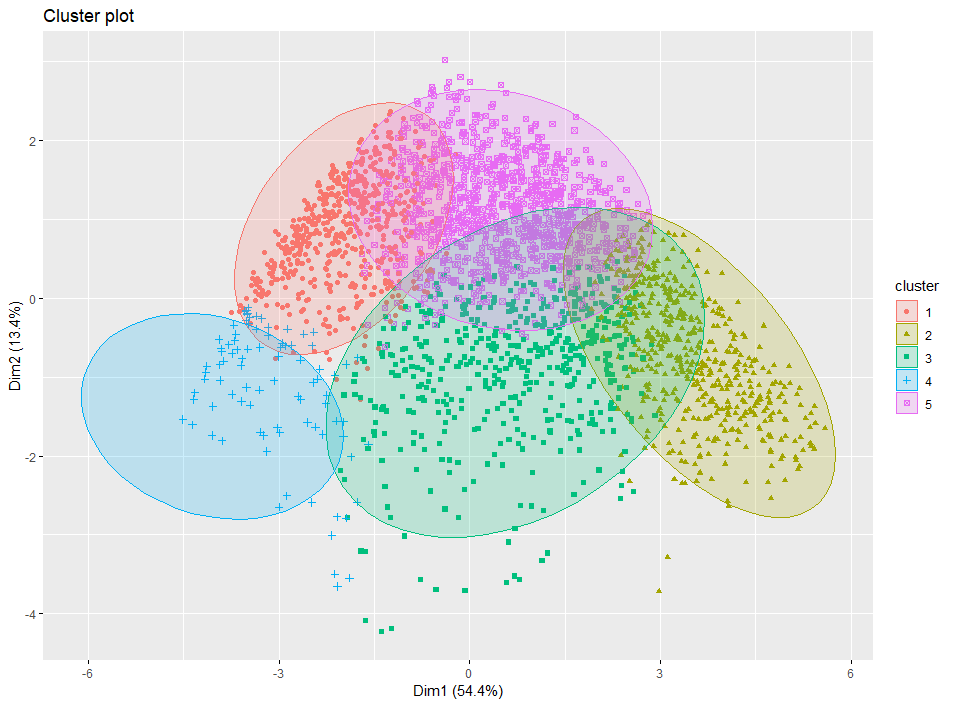


#CLARA

clara1<-clara(dat,k=2, metric="euclidean", samples=50,pamLike=TRUE, stand=FALSE)

#sampsize = min(n, 40 + 2 * k)

samples=integer, say N, the number of samples to be drawn from the dataset. The default, N = 5, is rather small for historical (and now back compatibility) reasons and we recommend to set samples an order of magnitude larger

stand= logical, indicating if the measurements in x are standardized before calculating the dissimilarities.


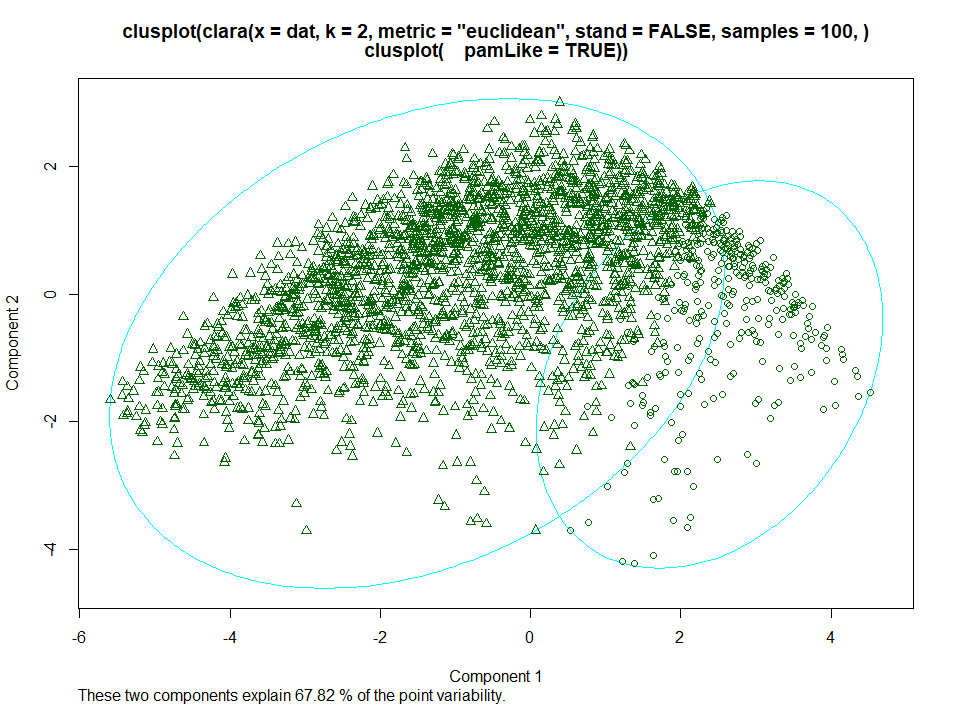

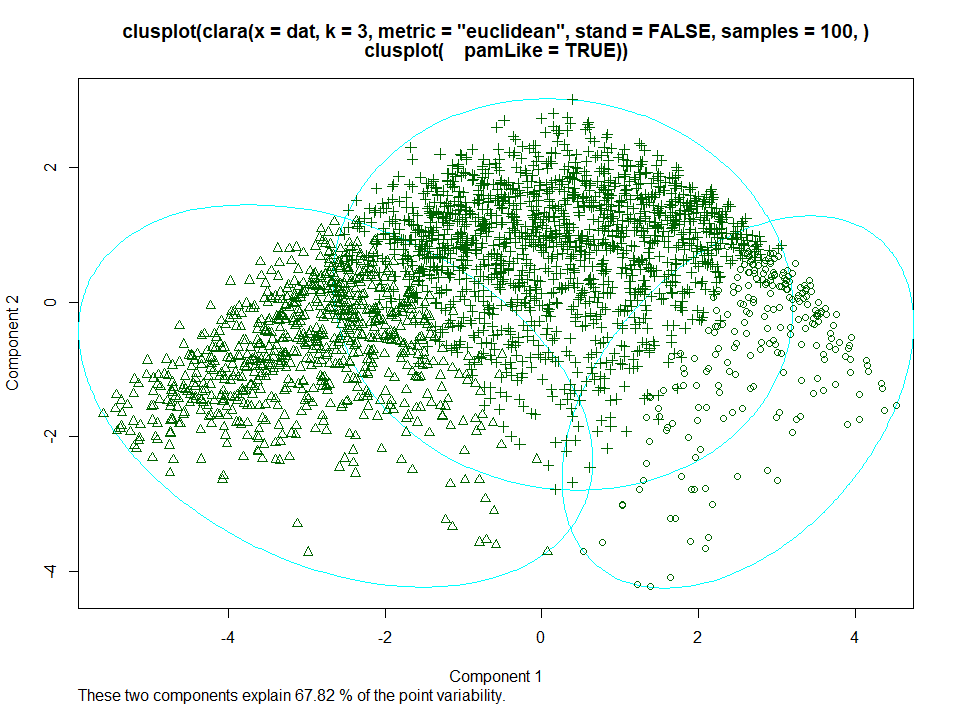


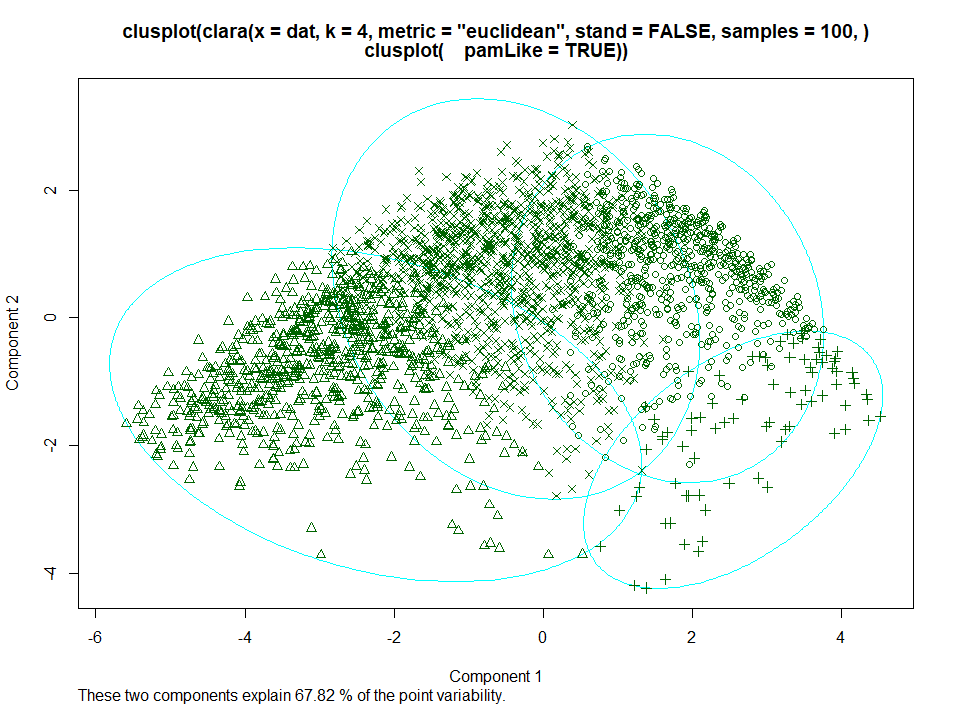

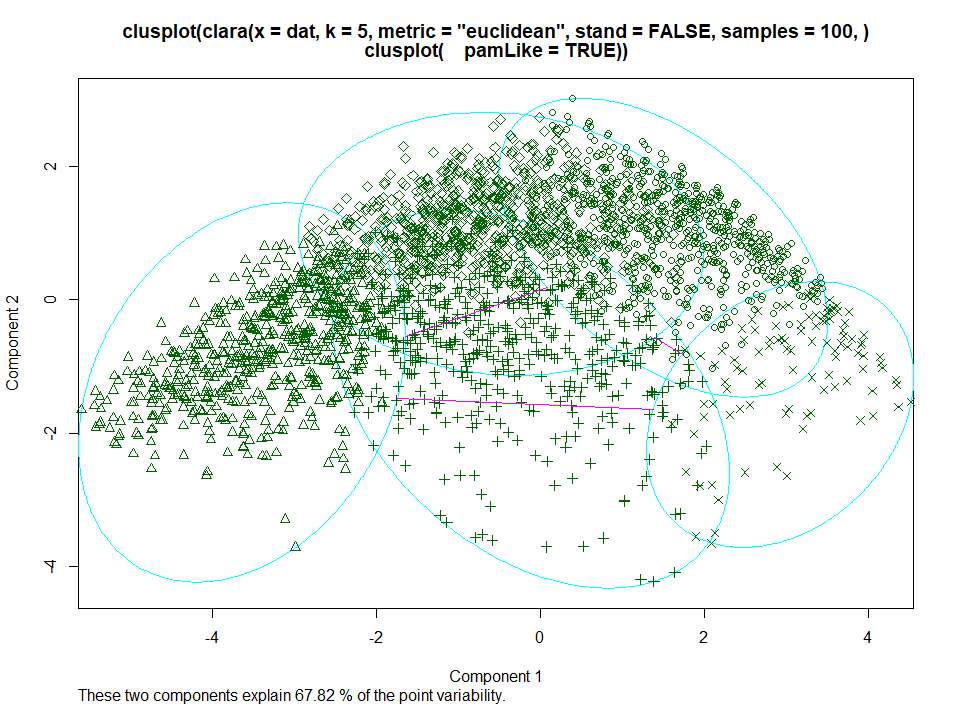


#MODEL-BASED (Expectation-Maximization)

library(mclust)

model <- Mclust(dat, 2)

dist.eu<-dist(dat, method = "euclidean", diag = FALSE, upper = FALSE, p = 2)

cs=cluster.stats(dist.eu,model$classification)

cs

plot(model, what = 'classification', main = FALSE)


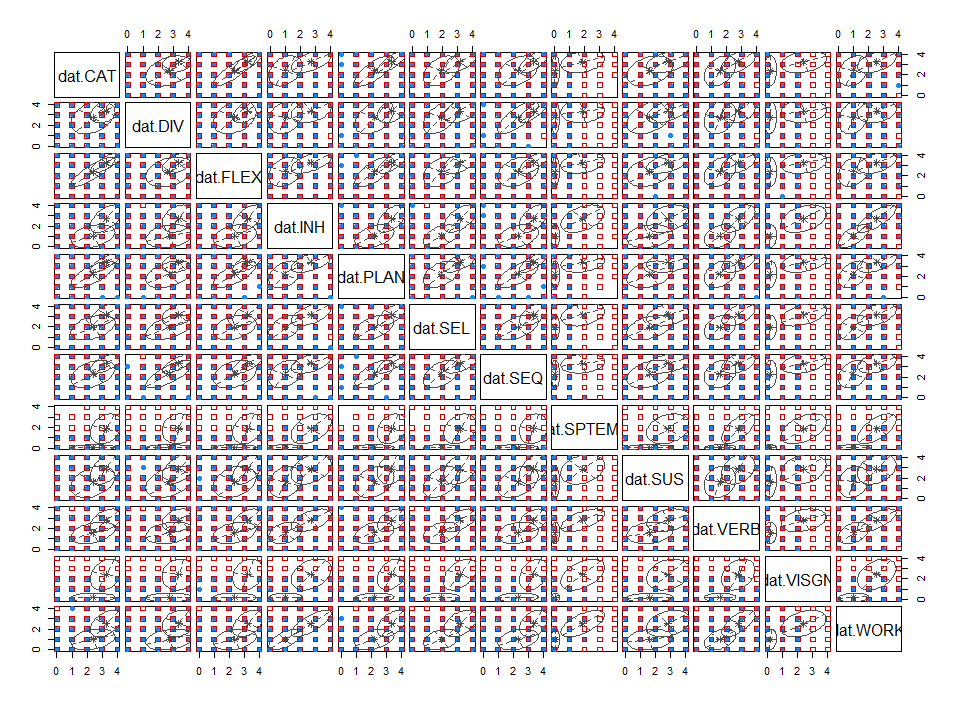


SOM (not mentioned in the manuscript, we have been trying different som groups)

library(kohonen)

som <- som(as.matrix(scale(dat)), somgrid(xdim=20, ydim=20, topo="hexagonal"))

groups<-3

model <- cutree(hclust(dist(som$codes[[1]])), groups)

# Colour palette definition

pretty_palette <- c("#1f77b4", '#ff7f0e', '#2ca02c', '#d62728', '#9467bd', '#8c564b', '#e377c2')

plot(som, type="mapping", bgcol = pretty_palette[model], main = "Clusters")

add.cluster.boundaries(som, model)

cc=cluster.stats(dist(som$codes[[1]]), model)

cc


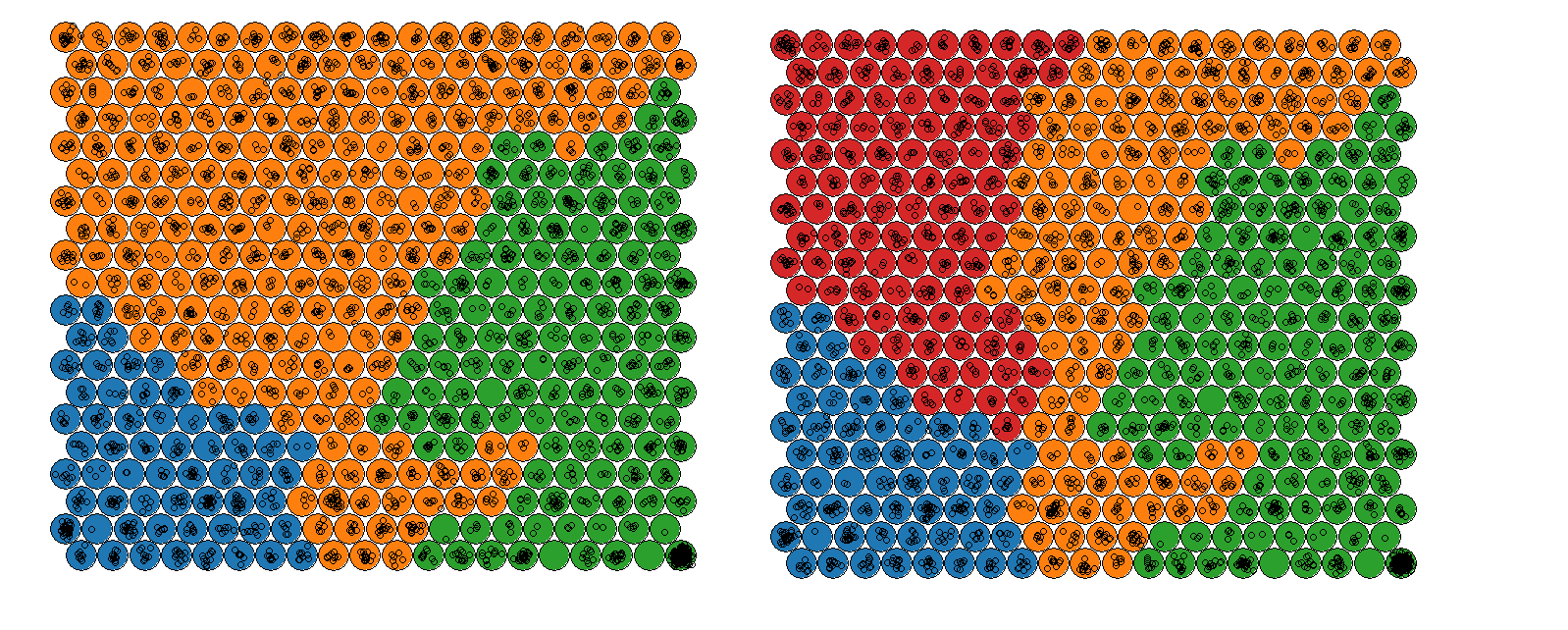


RANDOM FORESTS APPROACH

Table A3 confusion matrix generated from the supervised Random Forests

|  | **Class** | **1** | **2** | **3** |  |  | **Class Error** |
| --- | --- | --- | --- | --- | --- | --- | --- |
| k=3 | **1** | 478 | 4 | 0 |  |  | 0.0082 |
|  | **2** | 0 | 75 | 0 |  |  | 0.00 |
|  | **3** | 2 | 0 | 15 |  |  | 0.11 |
|  |  |  |  |  |  |  |  |
| k=4 |  | **1** | **2** | **3** | **4** |  |  |
|  | **1** | 382 | 9 | 5 | 0 |  | 0.035 |
|  | **2** | 6 | 80 | 0 | 0 |  | 0.069 |
|  | **3** | 0 | 0 | 75 | 0 |  | 0.00 |
|  | **4** | 2 | 0 | 0 | 15 |  | 0.11 |
|  |  |  |  |  |  |  |  |
| k=5 |  | **1** | **2** | **3** | **4** | **5** |  |
|  | **1** | 369 | 8 | 5 | 0 | 0 | 0.034 |
|  | **2** | 7 | 65 | 0 | 0 | 4 | 0.144 |
|  | **3** | 0 | 0 | 75 | 0 | 0 | 0.00 |
|  | **4** | 2 | 0 | 0 | 15 | 0 | 0.11 |
|  | **5** | 0 | 4 | 0 | 0 | 20 | 0.166 |


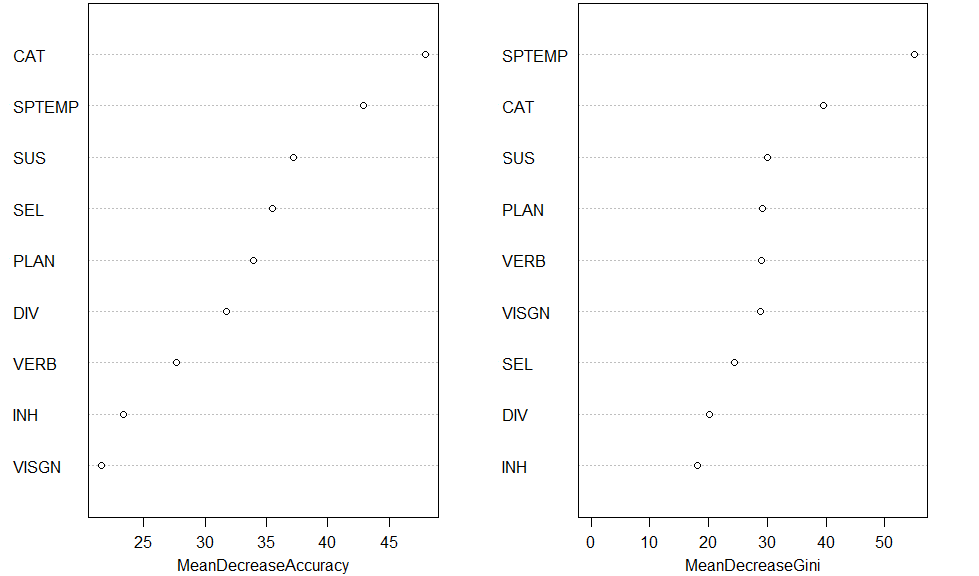


Figure A2. Variables importance ordered by Accuracy and Gini index

PCA

Table S1 shows the percentage of variance and the eigenvalues for the first 9 components of this matrix. The remaining components (31) correspond to a residual amount of variance. By selecting only the first three principal components, we reduce the dimensionality of the multivariate description so that the graphical representation and its subsequent interpretation are simplified.: The first component describes 55.04% of the variance, the second one describes 13.42% of the variance, and the third component describes 7.06% of the variance.

Table S1 Percentage of variance and the eigenvalues for the first 9 components

| **Comp** | **eigenvalue** | **Percentage of variance** | **Cumulative perc of variance** |
| --- | --- | --- | --- |
| 1 | 4.9542370 | 55.047078 | 55.04708 |
| 2 | 1.2086086 | 13.428984 | 68.47606 |
| 3 | 0.6354457 | 7.060508 | 75.53657 |
| 4 | 0.5831308 | 6.479231 | 82.01580 |
| 5 | 0.4898121 | 5.442357 | 87.45816 |
| 6 | 0.4537575 | 5.041750 | 92.49991 |
| 7 | 0.2576087 | 2.862319 | 95.36223 |
| 8 | 0.2256980 | 2.507756 | 97.86998 |
| 9 | 0.1917016 | 2.130018 | 100.00000 |

|  |
| --- |
| \|  \| \| --- \| |


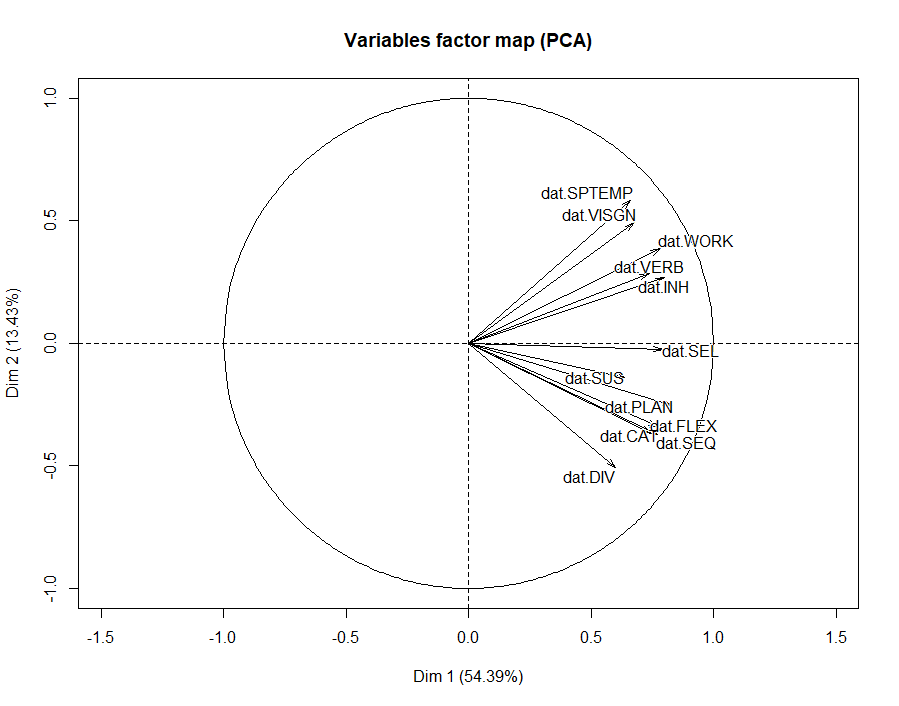


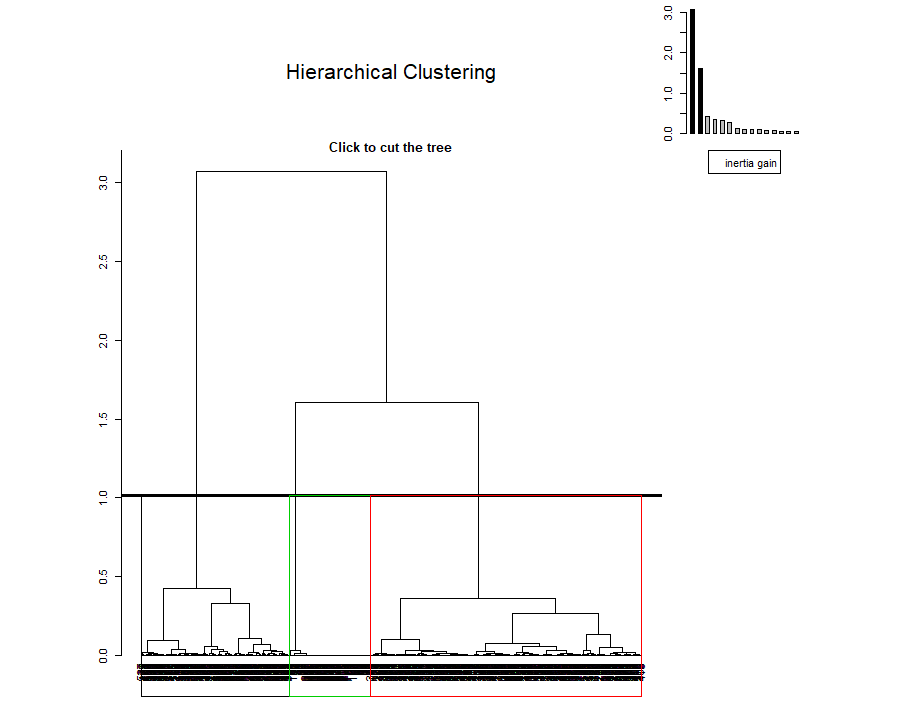


Figure A3. Three identified classes and gain decreasing after the 3^rd^ class (top right)


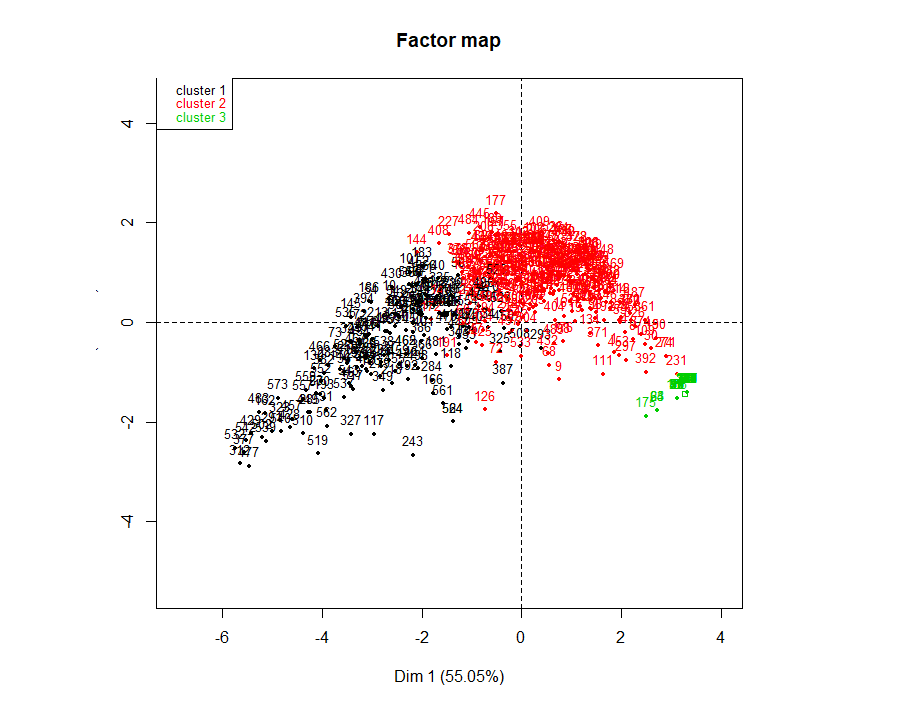


Figure S1. Obtained results in every task execution by cognitive function. Results are summarized weekly and plotted yearly during the whole period under study. Cluster1(blue) Cluster2(red)

For example patients started to execute Visual Gnosias tasks in 2014 and working memory tasks started in 2010 as shown in the next plot. Clearly the performance in visual gnosias tasks is lower in Cluster 2 patients.


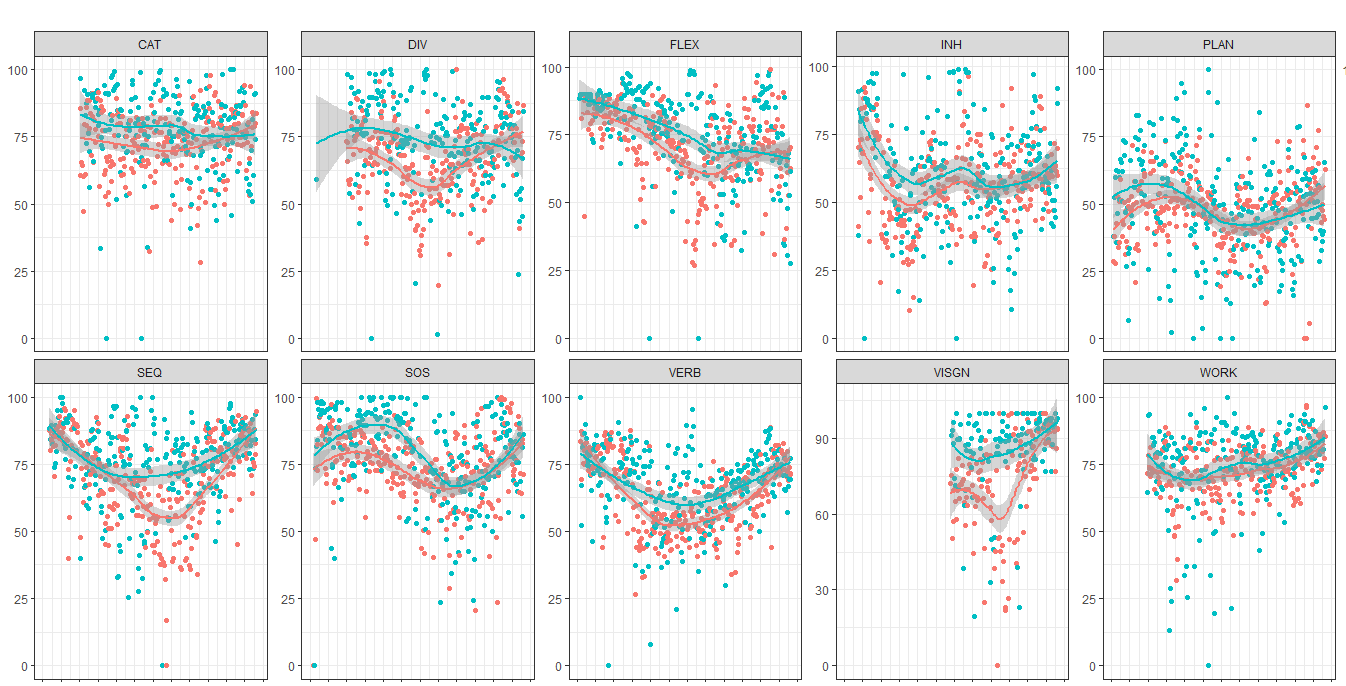


Figure S1. Results over time for cluster 1(blue) and cluster 2 (red) along time

Figure S2 Verbal tasks, visual gnosias and working memory tasks


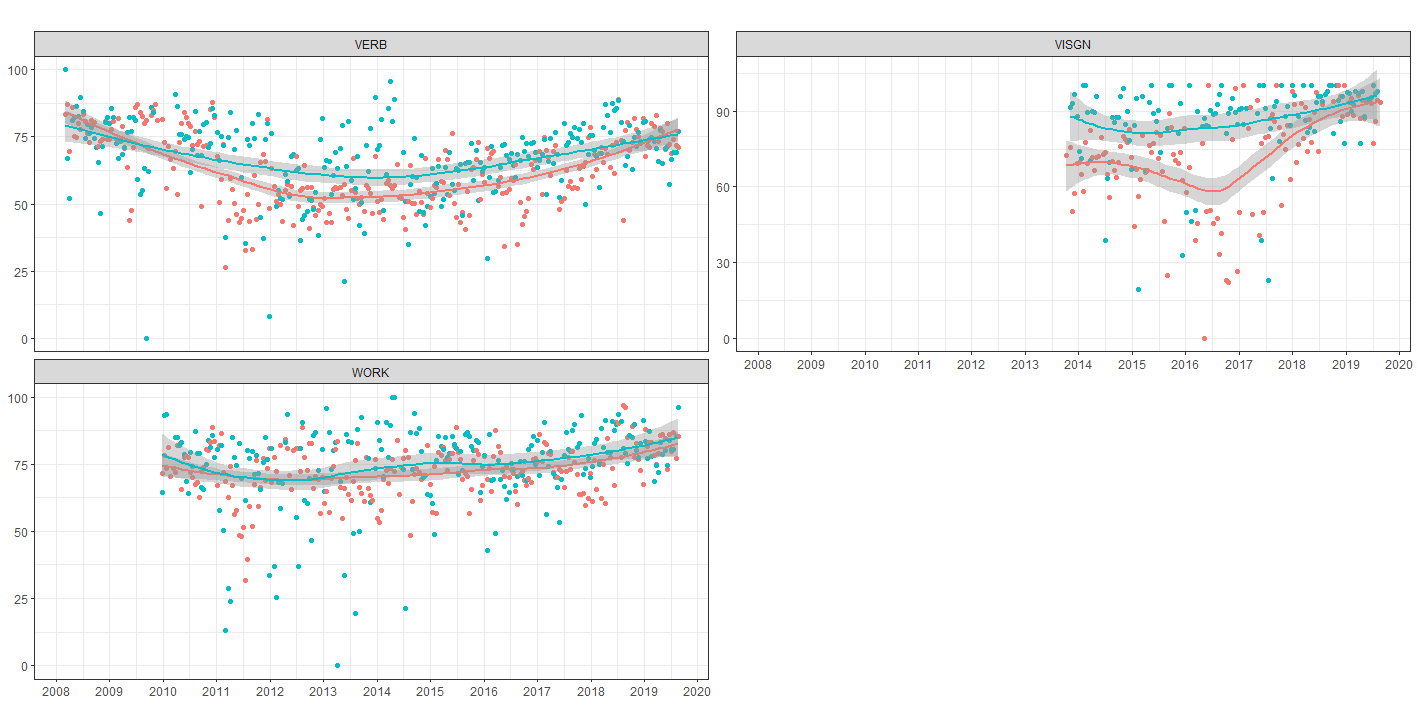


STROKE PATIENTS (n=323)

Initial correlation analysis


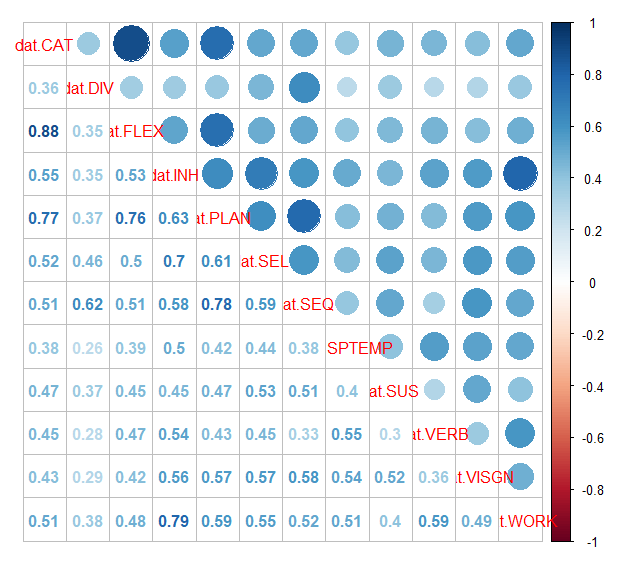

Supplement: Multimedia Appendix 1 [file medinform_v8i10e16077_app1.docx]
